# RASSF1A independence and early galectin‐1 upregulation in PIK3CA‐induced hepatocarcinogenesis: new therapeutic venues

**DOI:** 10.1002/1878-0261.13135

**Published:** 2021-11-20

**Authors:** Alexander Scheiter, Katja Evert, Lucas Reibenspies, Antonio Cigliano, Katharina Annweiler, Karolina Müller, Laura‐Maria‐Giovanna Pöhmerer, Hongwei Xu, Guofei Cui, Timo Itzel, Silvia Materna‐Reichelt, Andrea Coluccio, Kamran Honarnejad, Andreas Teufel, Christoph Brochhausen, Frank Dombrowski, Xin Chen, Matthias Evert, Diego F. Calvisi, Kirsten Utpatel

**Affiliations:** ^1^ Institute of Pathology University of Regensburg Germany; ^2^ Institute of Pathology University Medicine of Greifswald Germany; ^3^ Center for Clinical Studies University Hospital Regensburg Germany; ^4^ Department of Liver Surgery Center of Liver Transplantation West China Hospital of Sichuan University Chengdu China; ^5^ Department of Bioengineering and Therapeutic Sciences and Liver Center University of California San Francisco CA USA; ^6^ Division of Hepatology Department of Medicine II Medical Faculty Mannheim Heidelberg University Mannheim Germany; ^7^ Division of Personalized Tumor Therapy Fraunhofer Institute for Toxicology and Experimental Medicine Regensburg Germany

**Keywords:** alpelisib, galectin‐1, hepatocellular carcinoma, OTX008, SCD1, ZIP4

## Abstract

Aberrant activation of the phosphoinositide 3‐kinase (PI3K)/AKT/mTOR and Ras/mitogen‐activated protein kinase (MAPK) pathways is a hallmark of hepatocarcinogenesis. In a subset of hepatocellular carcinomas (HCCs), PI3K/AKT/mTOR signaling dysregulation depends on phosphatidylinositol‐4,5‐bisphosphate 3‐kinase, catalytic subunit alpha (PIK3CA) mutations, while RAS/MAPK activation is partly attributed to promoter methylation of the tumor suppressor Ras association domain‐containing protein 1 (RASSF1A). To evaluate a possible cocarcinogenic effect of PIK3CA activation and *RASSF1A* knockout, plasmids expressing oncogenic forms of PIK3CA (E545K or H1047R mutants) were delivered to the liver of *RASSF1A* knockout and wild‐type mice by hydrodynamic tail vein injection combined with sleeping beauty‐mediated somatic integration. Transfection of either PIK3CA E545K or H1047R mutants sufficed to induce HCCs in mice irrespective of *RASSF1A* mutational background. The related tumors displayed a lipogenic phenotype with upregulation of fatty acid synthase and stearoyl‐CoA desaturase‐1 (SCD1). Galectin‐1, which was commonly upregulated in preneoplastic lesions and tumors, emerged as a regulator of SCD1. Co‐inhibitory treatment with PIK3CA inhibitors and the galectin‐1 inhibitor OTX008 resulted in synergistic cytotoxicity in human HCC cell lines, suggesting novel therapeutic venues.

## Graphical abstract

Hydrodynamic tail vein injection of phosphatidylinositol‐4,5‐bisphosphate 3‐kinase, catalytic subunit alpha (PIK3CA) mutant forms E545K and H1047R induces stepwise hepatocarcinogenesis in mice, independent of Ras association domain‐containing protein 1 status. Gene expression analyses revealed an early increase in galectin‐1, which regulates the lipogenic enzyme stearoyl‐CoA desaturase‐1. PIK3CA‐ and galectin‐1 inhibitors act synergistically, pointing at novel therapeutic strategies.

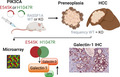

**Abbreviations**
ACAC: acetyl‐CoA carboxylase 1
ACLY: ATP citrate synthase
CK7: cytokeratin 7
COX2: prostaglandin‐endoperoxide synthase 2
CPS1: carbamoyl phosphate synthetase I
EMT: epithelial–mesenchymal transition
EOB: excess over the Bliss score
EV: empty vector
FASN: fatty acid synthase
FBXW7: F‐box and WD repeat domain‐containing 7
Gal‐1: galectin‐1
Gal‐3: galectin‐3
Gpc3: glypican‐3
GSEA: Gene Set Enrichment Analyses
HCC: hepatocellular carcinoma
IC50: half‐maximal inhibitory concentration
KO: knockout
MAPK: mitogen‐activated protein kinase
PI3K: phosphoinositide 3‐kinase
PIK3CA: phosphatidylinositol‐4,5‐bisphosphate 3‐kinase, catalytic subunit alpha
PIP_2_
: phosphatidylinositol (4,5)‐bisphosphate
PIP_3_
: phosphatidylinositol (3,4,5)‐trisphosphate
PPARγ: peroxisome proliferator‐activated receptor gamma
RASSF1A: Ras association domain‐containing protein 1
SCD1: stearoyl‐CoA desaturase‐1
SGK3: serum/glucocorticoid‐regulated kinase family member 3
STAT3: signal transducer and activator of transcription 3
WT: wild‐type
YAP: yes‐associated protein 1
ZIP4: Zrt‐Irt‐like protein 4

## 1 Introduction

Hepatocellular carcinoma (HCC) is the most frequent primary malignant tumor of the liver and a leading cause of cancer mortality worldwide [1]. North America and Europe experienced a constant rising incidence of HCC over the last decades [2]. Systemic therapeutic options for advanced disease stages are scarce, albeit the combination treatment of atezolizumab and bevacizumab is one recent glimmer of hope [3]. The pathogenesis of HCC is a multistep process involving the progression from cirrhosis to low‐grade dysplastic nodule, high‐grade dysplastic nodule, and, ultimately, HCC [4, 5]. The precise molecular mechanisms and their specific contributions to hepatocarcinogenesis remain poorly understood. Ras/mitogen‐activated protein kinase (MAPK) signaling is among the heavily implicated pathways in HCC, despite the absence of activating *RAS* mutations [6, 7]. Instead, diminished activity of Ras antagonists represents a plausible explanation for Ras/MAPK pathway unconstrained activity [8, 9, 10]. A critical physiological counterpart of Ras is Ras association domain‐containing protein 1 (RASSF1a), which is an established tumor suppressor embedded into an intricate regulatory network [11]. RASSF1A gained importance as it is frequently epigenetically silenced by aberrant hypermethylation in diverse types of cancer [12], including HCC [6, 13]. In addition to being a crucial negative‐feedback downstream effector of Ras Signaling [14], RASSF1A is a regulator of microtubule stability, Rho GTPases, and the Hippo pathway [15, 16, 17]. Thereby, multiple functions are exerted, such as cell cycle arrest, inhibition of migration, and metastasis through microtubular stabilization, reduction of epithelial–mesenchymal transition (EMT), and induction of apoptosis [18, 19, 20]. Interestingly, *RASSF1A* hypermethylation can be therapeutically reversed with panobinostat, a pan‐deacetylase inhibitor, which affects DNA methyltransferases' activity and expression, as demonstrated in a HepG2 xenograft model [21]. In HCC, transfection of *RASSF1A* yielded growth retardation in cell lines and the respective murine xenografts [22]. Furthermore, homozygous deletion of *RASSF1A* elicited late liver tumor susceptibility [23] and accelerated diethylnitrosamine‐induced HCC formation via autophagy promotion [24]. This body of evidence leaves little doubt on the oncogenicity of *RASSF1A* knockout (KO). However, the magnitude of the observed effects in the latter experimental models was somewhat limited, so that concurrent oncogenic stimuli are necessary together with *RASSF1A* loss to induce hepatocarcinogenesis.

Activation of the phosphoinositide 3‐kinase (PI3K)/RAC‐alpha serine/threonine‐protein kinase (AKT)/mTOR pathway could well qualify as a promising therapeutic candidate. Indeed, the PI3K cascade is one of the most deregulated pathways along tumorigenesis [14]. PI3K is a heterodimeric lipid kinase composed of a catalytic and a regulatory subunit (p110α or β or δ and p85α or β, respectively, for class Ia PI3K), which generates phosphatidylinositol (3,4,5)‐trisphosphate (PIP_3_) [25]. A pivotal downstream effector is AKT, which binds to PIP3 and is subsequently activated by phosphorylation. Once induced, AKT mediates various targets and regulates numerous cellular functions, such as cell cycle progression, growth, survival, and metabolism [26, 27]. Several genetic events triggering PI3K/AKT/mTOR activation have been reported [28]. Among them, activating mutations of the PI3K 110α subunit (*PIK3CA*) occupy a prominent position [29]. Important hot spot mutations include *PIK3CA* E542K, E545K, and H1047R, where the former affect the protein's helical domain and the latter its kinase domain [30, 31, 32]. The reported *PIK3CA* mutational frequency in human HCCs ranges from 4% to 6% [7, 33]. Murine models have only demonstrated a hepatic cocarcinogenic effect for activated PIK3CA in conjunction with either activated yes‐associated protein 1 (YAP), RasV12, or c‐Met to date [34, 35]. Indeed, injection of PIK3CA mutant forms alone promoted hepatic steatosis but did not induce hepatocarcinogenesis [35].

Here, we investigated a possible interplay between *RASSF1A* loss and the PI3K/AKT/mTOR pathway using an animal model of hydrodynamic tail vein injection with sleeping beauty‐mediated somatic integration. E545K and H1047R *PIK3CA* mutant forms were injected into homozygous *RASSF1A* KO mice. The resulting neoplastic lesions were subjected to a gene expression microarray analysis to shed light on PIK3CA‐driven hepatocarcinogenesis and identify putative therapeutic targets.

## 2 Materials and methods

### 2.1 Constructs and reagents

The constructs we applied in this study comprised pCMV/sleeping beauty transposase, pT3‐EF1α‐PIK3CA E545K, and pT3‐EF1α‐PIK3CA H1047R (including human PIK3CA clones), whose generation has been described previously [34]. pT3‐EF1α was used as empty vector (EV) control. For the production of lentiviruses, the plasmids psPAX2, pMD2.G, pLenti‐PIK3CA H1047R HA, and pLenti‐PIK3CA E545K HA were used. The plasmids were purified using the endotoxin‐free Maxi prep kit (Sigma‐Aldrich, St.Louis, MO, USA), after which the constructs were injected into the mice.

### 2.2 Mouse breeding and genotyping

A *RASSF1A* KO founder breeding pair was kindly provided by L. van der Weyden (Wellcome Trust Sanger Institute, Research Support Facility, Hinxton, Cambridge, CB10 1SA, UK). The genetic background of *RASSF1A* wild‐type (WT) and KO mice was C57BL/6J × 129Sv. *RASSF1A* WT mice were obtained by crossing *RASSF1A* KO mice with C57BL/6J mice purchased from Charles River Laboratories (Sulzfeld, Germany). Genotyping was performed on tail biopsies after DNA extraction. Polymerase chain reactions to detect the *RASSF1A* gene were carried out according to a previously established protocol [36]. The forward primer RSF‐5 (5′‐CTC GCC CCT GTC AGA CCT CAA TTT CCC‐3′) was applied together with the reverse primer RSF‐3 (5′‐CCA GGC TTC CTT CTC ACT CCT CTG CCG C3′), which yields a 400 base pair product in *RASSF1A* KO mice, where Exon 1 α has been deleted. The product length was evaluated by gel electrophoresis.

All experimental mice were kept and bred under standard conditions and stored in type III Makrolon cages with 12‐h light/dark cycles. The maximum number of mice was limited to five per cage. The animals were fed autoclaved food and water ad libitum.

### 2.3 Hydrodynamic injections, mouse monitoring, and tissue sampling

Male mice of 6–8 weeks were subjected to hydrodynamic tail vein injections in any of the following groups: untreated, 1× PBS, EV; pT3‐EF1α‐PIK3CA E545K, or pT3‐EF1α‐PIK3CA H1047R. Hydrodynamic tail vein injections were performed as described elsewhere [37]. Briefly, 10 µg of pT3‐EF1α‐PIK3CA, pT3‐EF1α‐PIK3CA H1047R, or pT3‐EF1α was combined with pCMV/sleeping beauty transposase in a ratio of 25 to 1 in a total volume of 2 mL 0.9% sodium chloride. The solution was filtered with 0.22‐µm mesh size. The total volume was injected into the lateral tail vein within 5–7 s. To attain at least 6–8 evaluable mice per time point and group, 10 animals per time point were injected in the experimental groups. Since control groups could be subjected to a combined analysis, only five animals were injected per condition and timepoint (Table 1).

**Table 1:** Overview of experimental groups and timepoints.

	1	2	3	4	5	6	7	8	9	10
	Experimental groups				Control groups					
Strain (*RASSF1A* KO or WT)	KO	KO	WT	WT	KO	WT	KO	WT	KO	WT
Injected construct	H1047R	E545K	H1047R	E545K						
1× PBS injection					X	X				
EV							X	X		
untreated									X	X
Animals per timepoint (*N*)										
1 week	10	10	10	10	5	5	5	5	5	5
1 month	10	10	10	10	5	5	5	5	5	5
3 months	10	10	10	10	5	5	5	5	5	5
6 months	10	10	10	10	5	5	5	5	5	5
9 months	10	10	10	10	5	5	5	5	5	5
12 months	10	10	10	10	5	5	5	5	5	5
Sum of animals per experimental group	60	60	60	60	30	30	30	30	30	30
Sum of all animals	420									

Animals were excluded from the evaluation if the injected total volume was below 2 mL. Another criterion to assess the quality of injection was the mouse behavior following the injection. Successfully injected mice displayed a decreased activity for at least 60 min due to the systemic volume challenge. Mice that did not show a similar behavior were excluded from the experiment. Mice were monitored daily and kept until the prespecified experimental time point. Respiratory distress, lethargy, and palpable liver masses equivalent to a size of ~ 3.5–4 cm were defined as termination criteria. The cervical dislocation was applied. A photodocumentation of the livers was performed, after which half of the livers was frozen in liquid nitrogen, and the other half was formalin‐fixed and paraffin‐embedded. 1–2 mm^3^ of liver tissue were fixed in glutaraldehyde. Animal breeding and animal experiments were in accordance with protocols by the Mecklenburg‐Western Pomeranian federal institution ‘Landesamt für Landwirtschaft, Lebensmittelsicherheit und Fischerei (LALLF) Mecklenburg‐Vorpommern’ (protocol number/Aktenzeichen: 7221.3‐1.1‐052/12).

### 2.4 Histology, immunohistochemistry, image acquisition, and proliferation index

At least two board‐certified pathologists and liver experts conducted the histopathological assessment of the liver lesions (KU, KE). The liver tissue was processed following the standard diagnostic institutional guidelines. Two‐micrometer‐thin histological sections were cut from formalin‐fixed, paraffin‐embedded tissue samples. The sections were deparaffinized through a series of xylene and gradient alcohols to water. For antigen retrieval, the slides were heated in a microwave oven for 10 min, while 10 mm sodium citrate buffer with a pH of 6.0 was applied. Afterward, the samples were cooled down to room temperature. The slides were treated with 1× Dako Peroxidase‐Blocking Solution® (cat S2023; Agilent Technologies Inc., Santa Clara, CA, USA) for 10 min. The primary antibody was diluted and applied in Dako Antibody Diluent® (cat. S2022, Agilent Technologies Inc.); the slides were placed in a humidity chamber and incubated at room temperature overnight (see Table 2 for a list of all primary antibodies). Following a subsequent washing step with Dako washing solution® (cat. S3006; Agilent Technologies Inc.), the secondary antibody Histofine Simple Stain MAX PO® anti‐goat or anti‐rabbit (Nacalai USA, Inc., San Diego, CA, USA) was administered for 60 min at room temperature. Two more washes in Dako washing solution® followed. For chromogenic reactions, we employed Dako Liquid DAB + Substrate Chromogen System® (cat. K346811‐2; Agilent Technologies Inc.), according to the manufacturer's instructions. Counterstaining was performed with Mayer's hemalum for 10 s. The application of coverslips was made automatically using Ventana BenchMark Ultra® (Roche, Penzberg, Germany).

**Table 2:** Antibodies used for immunohistochemistry and western blots.

Application	Dilution	Company	Catalog number
Immunohistochemistry			
p110α (PI3K)	1 : 100	Cell Signaling Technology, Inc.	4249
Phospho‐Akt (Ser473)	1 : 100	Cell Signaling Technology, Inc.	3787
COX2	1 : 300	Cell Signaling Technology, Inc.	12282
CPS1	1 : 100	Abcam, Cambridge, UK	ab129076
CK7	1 : 100	Abcam	ab181598
Desmin	1 : 1000	Abcam	Ab15200
pERK 1/2	1 : 100	Cell Signaling Technology, Inc.	4370
FASN	1 : 100	BD Biosciences, San Jose, CA, USA	610962
Gal‐1	1 : 100	Abcam plc.	ab138513
HA‐Tag	1 : 100	Cell Signaling Technology, Inc.	3724
Ki67	1 : 100	Bethyl Laboratories, Montgomery, TX, USA	IHC‐00375
SCD1	1 : 100	Cell Signaling Technology, Inc.	2794
a‐sma	1 : 200	Abcam plc.	ab5694
ZIP4	1 : 100	ProteinTech Group, Inc., Rosemont, IL, USA	20625‐1‐AP
Western blot			
ACAC	1 : 500	Cell Signaling Technology, Inc.	3676
ACLY	1 : 500	Cell Signaling Technology, Inc.	4332s
pAKT (S473)	1 : 1000	Cell Signaling Technology, Inc.	4060
pAKT (S473)	1 : 1000	ProteinTech Group, Inc.	66444‐1‐Ig
pAKT (S308)	1 : 1000	Cell Signaling Technology, Inc.	13038
t‐AKT	1 : 1000	Cell Signaling Technology, Inc.	9272
Pan‐AKT	1 : 1000	Cell Signaling Technology, Inc.	4691
COX2	1 : 1000	Cell Signaling Technology, Inc.	12282
pERK 1/2 (Thr202/Tyr204)	1 : 1000	Cell Signaling Technology, Inc.	4370
ERK1/2	1 : 1000	Cell Signaling Technology, Inc.	4695
FASN	1 : 1000	Santa Cruz Biotechnology, Inc.	sc‐55580
Gal‐1	1 : 1000	Abcam plc.	ab138513
Gal‐3	1 : 1000	Abcam plc.	ab76245
GAPDH	1 : 2000	Cell Signaling Technology, Inc.	5174
HA‐tag	1 : 1000	Cell Signaling Technology, Inc.	2367
PIK3CA	1 : 1000	Cell Signaling Technology, Inc.	4249
PPARγ	1 : 500	Cell Signaling Technology, Inc.	2435
SCD1	1 : 1000	Cell Signaling Technology, Inc.	2794
p‐SGK3 (Thr320)	1 : 1000	Cell Signaling Technology, Inc.	5642
SGK3	1 : 1000	Cell Signaling Technology, Inc.	8156
p‐STAT3 (Tyr705)	1 : 1000	Cell Signaling Technology, Inc.	9145
STAT3	1 : 1000	Cell Signaling Technology, Inc.	5904
ZIP4	1 : 1000	ProteinTech Group	20625‐1‐AP
β‐Actin	1 : 1000	Cell Signaling Technology	3700
HRP, goat anti‐mouse IgG (secondary antibody)	1 : 20 000	Abbkine Scientific Co., Ltd., Wuhan, China	A21010
HRP, goat anti‐rabbit IgG (secondary antibody)	1 : 20 000	Abbkine Scientific Co.	A21020

Images were acquired with the slide scanner Pannoramic 250 Flash III® (Sysmex, Kobe, Japan) with a 20× objective. Subsequently, images were displayed after stitching using the software CaseViewer® (Sysmex). With 300 pixels per inch, resolution screenshots were taken.

The stitched images of Ki67 immunohistochemistry were imported to the DeePathology™ STUDIO software (DeePathology.ai; Raanana, Israel). A board‐certified pathologist (KU) trained the algorithm to discern hepatocyte nuclei from nuclei of other cell types. These were determined as background cells (such as lymphocytes and Kupffer cells) and excluded from the analysis. Regions of interest were manually selected. A graphical representation of the calculation results was visually assessed for further validation.

### 2.5 Western blot analysis

For protein extraction, lysis of cells and mouse liver tissues was achieved using the Mammalian Protein Extraction Reagent (cat 78501; Thermo Fisher Scientific, Waltham, MA, USA) associated with the Halt Protease Inhibitor Cocktail (cat 78429; Thermo Fisher Scientific). An additional mechanical force was applied for tissue homogenization with the Next Advance Bullet Blender® Storm 24 (Next Advance, Inc.; Troy, NY, USA). The lysates were incubated for 30 min at 4 °C while vortexing every 5–10 min. A centrifugation step for 30 min (> 20 000 ***g***, 4 °C) ensued. Concentrations of total protein were assessed using the Bradford Protein Assay [38]. To attain a standard curve for linear regression, serial dilutions of BSA were employed.

For western blot analysis, protein lysates were separated by sodium dodecyl sulfate/polyacrylamide gel electrophoresis. 2.5 μg of total proteins was loaded onto BoltTM 4–12% Bis‐Tris Plus gels (Thermo Fisher Inc.) at 150 V for 30–60 min. Next, gels were incubated in 10% ethanol for dehydration before blotting the proteins to a membrane using the BlotTM 2 Gel Transfer Device (Thermo Fisher Inc.). After staining the membranes in Ponceau solution, they were placed in EveryBlot Blocking Buffer (Bio‐Rad Laboratories, Inc.; Hercules, CA, USA) for 15–30 min at room temperature. Primary antibodies (Table 2) were diluted in the blocking buffer and incubated overnight at 4 °C. The next day, membranes were washed in Tris‐buffered saline with Tween® (Cell Signaling Technology, Inc., Cambridge, UK) for 5 min at room temperature. Then, the secondary antibody was applied at room temperature for 1 h. The membranes were washed with Tris‐buffered saline with Tween®. The chemifluorescent signal was visualized by Clarity Max™ Western ECL Substrate (Bio‐Rad Laboratories) on a ChemiDoc™ MP Imaging System (Bio‐Rad Laboratories). For quantitative analysis of band intensities, version 6.1 of the software imagelab (Bio‐Rad Laboratories) was employed. Values were normalized to the corresponding β‐actin bands.

### 2.6 Transmission electron microscopy

Liver tissue samples were fixed in 0.1 m cacodylate‐buffered Karnovsky fixative containing 2.5% glutaraldehyde and 2% paraformaldehyde (Electron Microscopy Sciences, Hatfield, PA, USA) overnight at room temperature with a subsequent postfixation in 1% osmium tetroxide (Electron Microscopy Sciences), which was applied for 2 h. Next, the samples were dehydrated in graded ethanol (Sigma‐Aldrich). Afterward, they were embedded in an EMbed‐812 epoxy resin (Electron Microscopy Sciences). Following 2 days of heat polymerization at a temperature of 60 °C, 0.8 µm thin sections were prepared. These were stained with toluidine blue (Agar Scientific; Essex, UK) and basic fuchsine solution (Polysciences Inc.; Warrington, PA, USA). Subsequently, the epon block was adjusted to allow ultrathin sectioning. Eighty‐nm sections were cut with a diamond knife on a Reichert Ultracut‐S ultramicrotome (Leica, Wetzlar, Germany). These were double contrasted using aqueous 2% uranyl acetate (Honeywell International Inc., Morristown, NJ, USA) and lead citrate solutions (Leica) for 10 min each. A LEO912AB transmission electron microscope (Zeiss, Oberkochen, Germany) operated at 100 kV was used for imaging the ultrathin sections.

### 2.7 Gene expression microarray hybridization and analysis

For comparative microarray of mouse livers, the RNA was isolated from liver tissue using the NucleoSpin® RNA Plus Kit (Macherey‐Nagel GmbH & Co. KG, Düren, Germany), following the manufacturer's instructions. Gene expression microarray analysis was performed with mouse Sentrix® BeadChips (Illumina, San Diego, CA, USA). We followed the workflow established by the manufacturer. Briefly, cDNA synthesis, *in vitro* transcription, and cleanup were conducted using the Ambion® RNA Amplification Kit (Illumina). The samples were quantified after adding Molecular Probes Ribo Green® (Thermo Fisher Scientific) by fluorometric measurement. Afterward, the samples were hybridized, and an image was extracted with the BeadArray Reader (Illumina). The data were analyzed with the BeadStudio® application (Illumina).

Raw data were background corrected, quantile‐normalized, and log2‐transformed using the limma package [39] provided by R/Bioconductor (https://www.bioconductor.org). Subsequently, differentially expressed genes were calculated, using the limma package. Concerning multiple testing, Benjamini and Hochberg's method was applied to adjust the *P*‐values. Heatmaps were generated with the pheatmap package provided by R/Bioconductor (https://www.bioconductor.org). Furthermore, preranked Gene Set Enrichment Analysis (GSEA; Hallmark gene sets) was performed using the official software tools from the Broad Institute (Boston, MA, USA, https://www.gsea‐msigdb.org) [40]. For this purpose, all probes representing the same gene symbol were averaged.

For comparative microarray of stably transfected HCC cell lines, the sample preparation for microarray hybridization was conducted as described in the Applied Biosystems™ GeneChip™ Whole Transcript (WT) PLUS Reagent Kit User Guide (Thermo Fisher Scientific). In brief, 200 ng of total RNA was used to generate double‐stranded cDNA. Twelve microgram of subsequently synthesized cRNA was purified and reverse transcribed into single‐stranded (ss) cDNA, with unnatural dUTP residues incorporated. Purified ss cDNA was fragmented using uracil DNA glycosylase (UDG) and apurinic/apyrimidinic endonuclease 1 (APE 1) followed by terminal labeling with biotin. Subsequently, 3.8 µg of fragmented and labeled ss cDNA was hybridized to Applied Biosystems™ GeneChip™ Clariom S human arrays for 16 h at 45 °C and 60 r.p.m. in an Applied Biosystems™ GeneChip™ hybridization oven 640. Hybridized arrays were washed and stained in an Applied Biosystems™ GeneChip™ Fluidics Station FS450, and the fluorescent signals were measured with an Applied Biosystems™ GeneChip™ GeneChip Scanner 3000 7G System. Fluidics and scan functions were controlled by the Applied Biosystems™ GeneChip™ Command console v5.0 software (Thermo Fisher Scientific Inc.).

Sample processing was performed at a Genomics Core Facility, ‘KFB—Center of Excellence for Fluorescent Bioanalytics’ (Regensburg, Germany; www.kfb‐regensburg.de). For the data analysis, summarized probe set signals in log_2_ scale were calculated by using the GCCN‐SST‐RMA algorithm with the Applied Biosystems™ GeneChip™ Expression console v1.4 Software. After exporting into Microsoft Excel comparison, fold changes were calculated. Probe sets with a fold change above 2.0 fold were considered significantly regulated.

Heatmaps were generated with the Transcriptome Analysis Console (Applied Biosystems, Waltham, MA, USA). GSEA analysis was performed using the WEB‐based GEne SeT AnaLysis Toolkit [41] by selecting the Panther pathway functional database.

### 2.8 Cell culture and *in vitro* studies

The human HCC cell lines PLC/PRF/5, HLE, HLF, Snu182, Snu387, and Snu449 were cultured in 5% CO_2_ at 37 °C in a humidified incubator. Cell lines were purchased from ATCC (Manassas, VA, USA). Cells were grown in Dulbecco's modified Eagle medium (DMEM; Gibco, Grand Island, NY, USA) or RPMI 1640 Medium (Gibco) supplemented with 5% FBS (Gibco), 100 mg·mL^−1^ streptomycin, and 100 U·mL^−1^ penicillin.

We performed cell viability assays using the xCELLigence® real‐time cell analysis dual plate (RTCA DP) device (OLS OMNI Life Science GmbH & Co KG; Bremen, Germany). For impedance‐based real‐time cell index measurement, cells were grown on E‐Plate 16 PET (Agilent Technologies, Inc.). Measurement sweeps were acquired every 15 min. Six thousand two hundred fifty cells suspended in a total volume of 150 µL of growth medium were seeded in each well. After 24 h, varying concentrations of dimethyl sulfoxide (Sigma‐Aldrich), the inhibitory compounds alpelisib (MedChemExpress; LLC., Monmouth, NJ, USA), and/or OTX008 (MedChemExpress) were added. Afterward, the measurement was acquired for a total of 72 h. Raw data were analyzed with the rtca software (OLS OMNI Life Science GmbH & Co KG). The data were normalized to the timepoint of inhibitor addition. Synergism was evaluated 24 h after adding the inhibitory compounds using the software compusyn (ComboSyn, Inc., Paramus, NJ, USA), which generates dose–effect curves and isobolograms, and determines the combination index.

For alpelisib single treatment, cells were seeded into 6‐well plates at a density of 3–5 × 10^5^ cells in 2 mL medium per well. The next day, either alpelisib in varying concentrations or matched DMSO was added. After an incubation of 48 h, cells were harvested, centrifuged, and the cell pellet was used for further processing.

For galectin‐1 (Gal‐1) silencing, cells were seeded at a density of 3 × 10^5^ cells in 2 mL of medium per well in six‐well plates. Cells were transfected with Silencer® Select Negative Control #1 siRNA (Thermo Fisher Inc) or LGALS1 siRNA (Eurofins Genomics, Ebersberg, Germany) with the sense sequence 5′‐[UUGCUGUUGCACACGAUGGUGUUGG]‐3′ the following day using Lipofectamine® RNAiMAX (Thermo Fisher Inc) according to the manufacturer's instructions. Lipofectamine and siRNA were diluted in OptiMEM® Reduced Serum Medium (Thermo Fisher Inc.) and combined. Medium in the wells was discarded, and cells were washed with 1× PBS before adding the transfection solution. After an incubation period of 48 h, the transfection was repeated once more. Cells were harvested after an additional 24 h of incubation using cell scrapers. Harvested cell suspensions were centrifuged (300g, 5 min). The pelleted cells were used for further analyses.

For OTX008 treatments, cells were seeded at a density of 3 × 10^5^ cells in 2 mL of medium per well in six‐well plates. The next day, OTX008 (or an adjusted amount of DMSO) was added to a total concentration of 20 µm. After an incubation period of 48 h, cells were harvested using cell scrapers. Harvested cell suspensions were centrifuged (300 ***g***, 5 min). The pelleted cells were used for subsequent western blot analysis.

HEK‐293FT cells were used for producing lentiviral particles. They were plated in the 10‐cm dish and cultured in a DMEM without PS at 37 °C, 5% CO_2_. After the HEK‐293FT cells reached to 60–70% confluence, they were cotransfected with the plasmids mixture (9.2 μg psPAX2 + 2.8 μg pMD2.G + 12 μg pLenti‐PIK3CA H1047R/E545K/EGFP) and 30 μL Lipofectamine 2000 reagent (Invitrogen) diluted in 500 μL OptiMEM for 72 h. Lentiviral supernatant was then harvested and filtered through a 0.45‐mm PES filter (Millipore, Bedford, MA, USA). Subsequently, SNU387 and SNU449 cells were infected with the virus and fresh Roswell Park Memorial Institute 1640 (RPMI 1640) medium at the volume ratio of 1 : 1. After 48 h of transfection, cells were treated with puromycin‐containing media (1.5 μg·mL^−1^) to select cells with stable expression of the target gene with the puromycin resistance.

To determine the half‐maximal inhibitory concentration (IC50) values for OTX008 and alpelisib and perform combination treatments in the stably transfected cell lines, these were seeded into 24‐well plates at 2.5 × 10^5^ and treated with escalating concentrations of alpelisib/OTX008 or both compounds for 48 h. Subsequently, the cells were washed in PBS 3 times, followed by crystal violet staining for 10 min. After washing, the stained cells were incubated in lysis solution for 20 min. Next, the diluted solution was added to 96‐well plates to measure OD values at 590 nm by the BioTek ELX808 Absorbance Microplate Reader (Thermo Fisher Scientific). The IC50 values were calculated based on the OD values. These experiments were repeated in triplicates.

### 2.9 ATP detection assay and drug screening

PLC/PRF/5 cells were seeded into 384‐well plates (µClear #781091, Greiner Bio‐One GmbH, Frickenhausen, Germany) at a density of 2500 cells per well. Cells were treated at the time of seeding with the set concentration of OTX008 20 µm (#35318, MedChemExpress), alpelisib 1 µm (S2814, Selleck Chemicals LLC, Houston, TX, USA), buparlisib 1 µm (S2247, Selleck Chemicals LLC), or taselisib 1 µm (S7103, Selleck Chemicals LLC) either alone or in combination using the D300e digital dispenser (Tecan Group, Männedorf, Switzerland). Cell viability was measured 72 h after treatment using an ATPlite 1 step detection assay (#6016739, PerkinElmer, Inc., Waltham, MA, USA). To assess synergy between OTX008 and the PI3K inhibitors, we used the Bliss model of independence [42] to calculate the combined treatment's expected effect, assuming the compounds act independently.

Expected_AB_ = E_A_ + E_B_ − EA_EB_

E_A_ and E_B_ are the effects of the two compounds in monotherapy, measured as the reduction in viability. According to the Bliss model, the observed effect of the combination therapy is directly compared with the expected effect and calculated as the excess over the Bliss score (EOB) [43].

EOB = Observed_AB_ − Expected_AB_

If EOB is > 0, the combined compounds have an effect more potent than if they acted independently.

A compound library including 315 approved anticancer drugs was purchased from TargetMol (L2110; Target Molecule Corp., Boston, MA, USA), diluted to a concentration of 1 mm, and 40 nL was dispensed in 384‐well plates (Greiner µClear #781091, Greiner Bio‐One GmbH) using an acoustic liquid handler (Echo® 550, Beckman Coulter Life Sciences, Brea, CA, USA). PLC/PRF/5 cells were detached and separated in two tubes containing either OTX008 at a final concentration of 20 µm or DMSO vehicle. The cells were then seeded using a liquid dispenser (Multidrop™ Combi, Thermo Fisher Scientific) at 2500 cells per well in prespotted assay plates at a final volume of 40 µL per well to reach a final concentration of 1 µm of the compounds. Cells were incubated at 37 °C, 5% CO_2_ for 72 h. At the end of the incubation time, 10 µL of a solution of PBS and Hoechst 33342 (2 drops·mL^−1^, NucBlue™ Live ReadyProbes™ Reagent; Thermo Fisher Scientific) was added to each well using a liquid dispenser (Multidrop™ Combi; Thermo Fisher Scientific), incubated for 1 hour and imaged for brightfield and Hoechst staining using the Operetta CLS™ High‐Content Analysis System (PerkinElmer, Inc.). Stained nuclei were counted using the Harmony® software (PerkinElmer, Inc.), and the number of nuclei measured cell viability and proliferation. For each plate, the proteasome inhibitor Carfilzomib was used as a positive control and DMSO as a negative control to calculate the *Z*′ value as a plate QC criteria: All plates had a *Z*′ > 0.7, above the widely accepted threshold of 0.5. Data from two independent experiments were averaged (correlation coefficient between replicates 0.96). The number of nuclei in each well was normalized to DMSO‐treated controls, and normalized viability was used to calculate the EOB as described above. Compounds were defined as a hit if the observed normalized viability upon treatment in combination with OTX008 was below 0.6 and the *Z* score of EOB was > 1.5. Information regarding the mode of action and the targeted genes for each compound was obtained from the drug repurposing hub database [44].

### 2.10 Statistical analyses

Descriptive statistics compared tumor occurrence frequency. Comparisons between two groups were conducted with nonparametric Mann–Whitney *U*‐tests due to small sample sizes (quantification of lipogenic enzymes in Gal‐1 silencing experiments). For multiple comparisons, nonparametric data were compared using the Kruskal–Wallis test without adjustment (liver/weight over bodyweight comparison; quantification of western blots for Gal‐1 in PIK3CA E545K liver lysates; quantification of western blots in alpelisib treatment experiments).

Kaplan–Meier curves and log‐rank tests were used to compare survival between *RASSF1A* WT and KO mice with PIK3CA E545K injection.

A mixed linear model (maximum‐likelihood estimation, unstructured repeated covariance type) was applied to evaluate repeated proliferation measures between *RASSF1A* KO and WT mice and assess the proliferation within normal tissue, preneoplastic lesions (PREs), and tumors. Moreover, corresponding injection groups were compared against each other separately in *RASSF1A* WT versus KO mice.

graphpad prism version 9, graphpad prism r version 4.0.3 (GraphPad Software; San Diego, CA, USA), and spss version 26 (IBM; Armonk, NY, USA) were employed. All *P*‐values were obtained in two‐tailed tests, and *P* ≤ 0.05 was considered statistically significant.

Microarray data were analyzed using the r package Limma. Moderated contrast *t*‐tests were computed, and Benjamini and Hochberg's method to adjust for multiple testing was applied to microarray expression data [45, 46].

## 3 Results

### 3.1 PIK3CA mutant forms E545K and H1047R cause hepatocarcinogenesis irrespective of *RASSF1A* mutational background

**Fig. 1:**
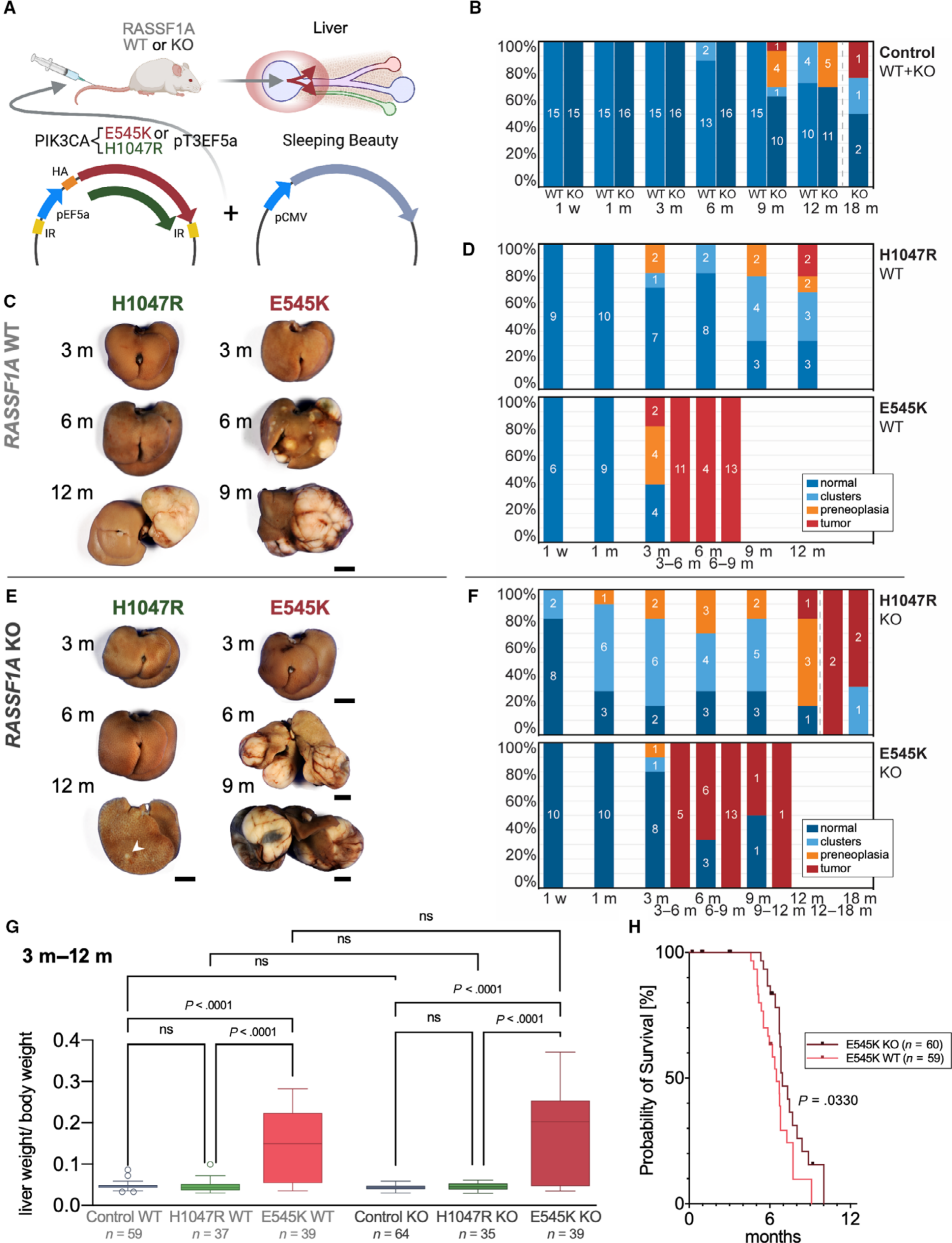
Hydrodynamic injection of *PIK3CA*‐mutated forms in the mouse liver drives hepatocarcinogenesis irrespective of *RASSF1A* background. (A) Scheme of hydrodynamic tail vein injections of PIK3CA mutant forms E545K or H1047R in conjunction with a plasmid encoding sleeping beauty transposase into either *RASSF1A* WT or KO mice leading to preferential transfection of pericentral hepatocytes as indicated by the red circle and arrows in the liver scheme. (B) Stacked bar charts showing the occurrence frequency of clear‐cell clusters, PREs, and tumors as well as normal‐appearing liver tissue (specified in the legend) at noted time points based on histological examination in *RASSF1A* WT versus KO mice in combined injection control groups (untreated, PBS‐injected, and EV‐injected mice). Central digits specify the number of observed animals in the respective classification subgroups. The dashed line is indicative of an extended observation period for four *RASSF1A* KO mice. (C) Representative gross images of livers of *RASSF1A* WT mice injected with *PIK3CA* mutant forms H1047R and E545K at indicated time points. Scale bar: 0.5 cm. (D) Stacked bar charts visualizing the frequency of occurrence of neoplastic lesions (specified in the legend) in *PIK3CA H1047R* and E545K‐injected *RASSF1A* WT mice. (E) Representative gross images of livers of *RASSF1A* KO mice injected with *PIK3CA* mutant forms H1047R and E545K at noted time points. The arrowhead points at a small tumor. Scale bars: 0.5 cm. (F) Stacked bar charts visualizing the frequency of occurrence of neoplastic lesions (specified in the legend) in *PIK3CA* H1047R and E545K‐injected *RASSF1A* KO mice at noted time points. (G) Diagram comparing the ratio of liver weight and body weight as tumor burden surrogate for combined time points ranging from 3 to 12 months in the respective *PIK3CA* injection groups sorted by *RASSF1A* background. Tukey method box plots are displayed. A Kruskal–Wallis test was calculated. (H) Kaplan–Meier survival curves (euthanasia based on termination criteria) of *RASSF1A* WT and KO mice from *PIK3CA* E545K injection group. Log‐rank test showed a minor significant longer survival time in KO than WT mice (*P* = 0.0330). Mice number in each arm is labeled in the figure legend.

A mouse model of hydrodynamic tail vein injection with sleeping beauty‐mediated somatic integration [47, 48, 49, 50] was chosen to evaluate *RASSF1A* and *PIK3CA* mutant forms carcinogenic cooperativity. This procedure favors the transfection of pericentral hepatocytes (acinus zone 3) and yields transfection efficiencies in the range of 5–10% of hepatocytes (Fig. 1A) [51].

Contrary to our expectations from previous injections of mutant *PIK3CA* containing plasmids [34, 35], mice in all PIK3CA injection groups developed liver tumors within the prespecified observation period of 12 months. This unexpected finding presumably depended on the mixed background used for the experiments (C57BL/6J × 129Sv), which differs from the previously employed FVB/N inbred mouse strain [35].

Based on combined gross and histological examination, the observed lesions were stratified into the following categories: preneoplastic lipid‐rich clusters, preneoplasias (criteria of expansive growth with initial compression of surrounding tissue (SR) and estimated cell content > 100), and tumors (irregular borders, presence of necrosis, expansive growth with evident compression or diffuse infiltration of SR, macroscopic correlation, cytologic signs of malignancy). Tumors in *PIK3CA* E545K‐injected mice were already detectable after 3 months, instead of a 12‐month latency of tumorigenesis in the *PIK3CA* H1047R injection groups. Surprisingly, H1047R injections yielded discernible tumors in only three of 112 mice within the defined observation time of 12 months. In contrast, E545K injections resulted in numerous tumors (tumors in 56 of 113 mice), which frequently necessitated a premature termination. Serving as solid evidence against cooperativity, neither clusters, PREs, nor tumors displayed a marked difference in the occurrence frequency when comparing PIK3CA mutant forms injected in *RASSF1A* WT and *RASSF1A* KO mice. A combined control group (~ 5 mice each without transfection, injection of 1× PBS, and transfection of the EV) did not demonstrate spontaneous tumorigenesis in *rassf1a* WT mice. However, the *RASSF1A* KO mouse control group developed a total of two tumors at the experimental time points of 9 and 18 months (extended period of observation; Fig. 1B–F ).

To assess differences in tumor burden between experimental groups, the liver weight/body weight ratio for the combined experimental time points ranging from 3 to 12 months was compared using the Kruskal–Wallis test (Fig. 1G). The *post hoc* test showed that PIK3CA E545K had increased oncogenic potency compared to H1047R and control in *RASSF1A* WT and KO mice (*P*‐values < 0.0001). PIK3CA H1047R‐injected mice were neither significantly different from control in *RASSF1A* WT nor KO mice (*P*‐values > 0.05). Moreover, the lack of significant differences between *RASSF1A* KO and WT mice substantiated that *RASSF1A* loss does not increase tumor burden (*P*‐values > 0.05).

Next, we conducted a survival analysis in mice with only tumor‐related deaths in *PIK3CA* E545K subgroups. Animals were deliberately euthanized based on the assessment of the following termination criteria: respiratory distress, lethargy, and palpable liver masses equivalent to a size of ~ 3.5–4 cm. Premature termination occurred at the formerly denoted time points 3–6, 6–9, and 9–12 months. A minor, more prolonged survival of *PIK3CA* E545K‐injected mice was detected in *RASSF1A* KO mice (median survival = 6.9 months) compared with *RASSF1A* WT mice (median survival = 6.5 months, *P* = 0.0330; Fig. 1H).

Altogether, these data indicate that *RASSF1A* loss does not increase the susceptibility to *PIK3CA* mutant form‐mediated hepatocarcinogenesis and even shows a tendency to improve survival in the *PIK3CA* E545K group. Of note, the only two tumors observed in the control group emerged in *RASSF1A* KO mice. Even if these tumors were indeed *RASSF1A* related, a very long latency was required for tumorigenicity.

**Fig. 2:**
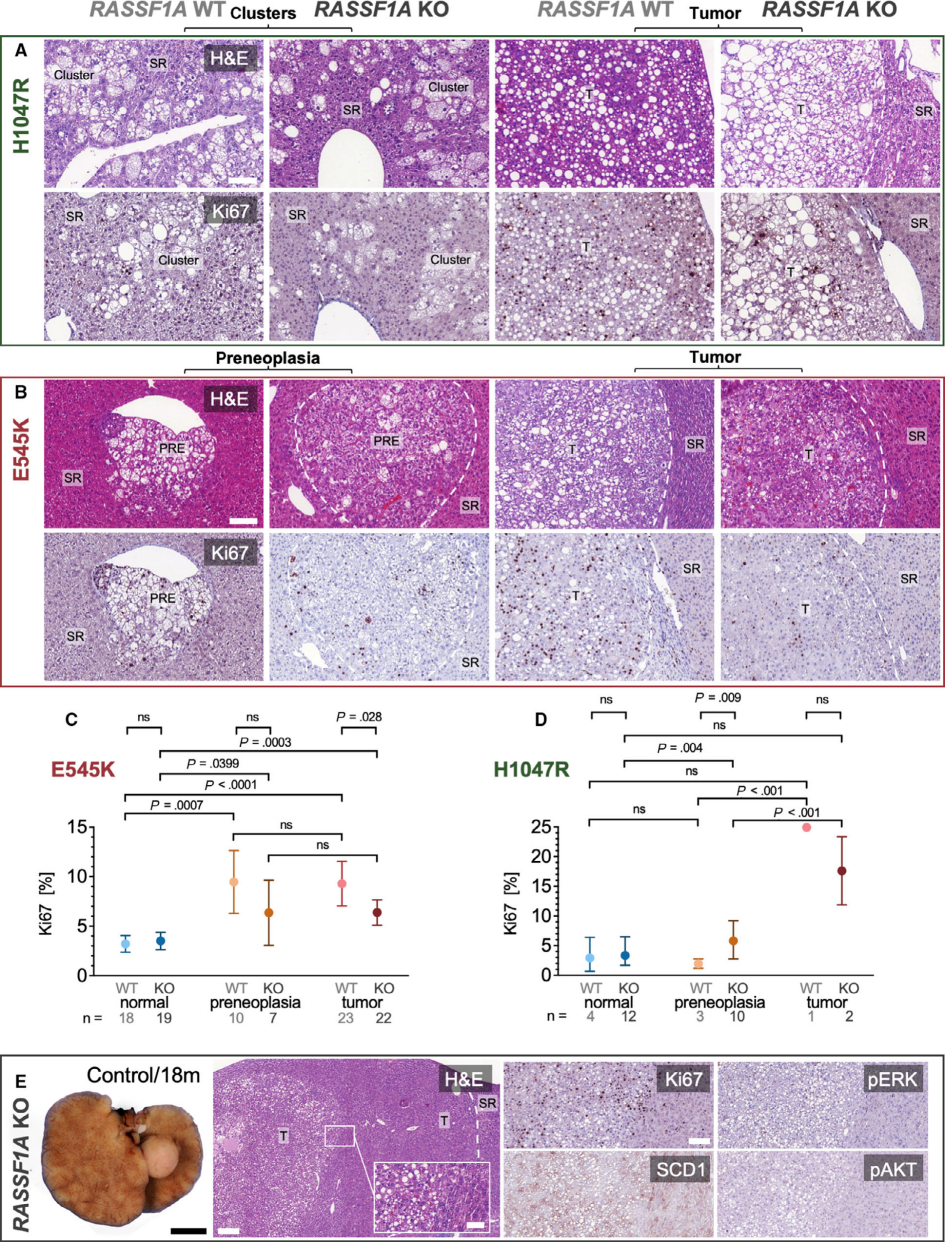
Histology and proliferation in *PIK3CA*‐dependent tumors induced in *RASSF1A* WT and KO mice. (A) Representative histological images of pericentral lipid‐rich clusters and induced lipid‐rich tumors resulting from *PIK3CA* H1047R injections in *RASSF1A* WT and KO mice. The number of animals displaying these clusters can be inferred from Fig. 1D,F. Scale bar: 100 µm. (B) Histological sections of *PIK3CA* E545K‐induced PREs and tumors in *RASSF1A* WT and KO mice with corresponding immunohistochemical staining for Ki67. The number of stained animals can be inferred from (C) and (D). Scale bars: 100 µm. (C) Quantification of Ki67 proliferation index in *RASSF1A* WT and KO mice in normal‐appearing liver tissue, PREs, and tumors in *PIK3CA* E545K‐injected mice; *n*(mice, KO) = 22; *n*(mice, WT) = 24. Visualization as estimated mean with 95% confidence interval. A mixed linear model was used. (D) Quantification of Ki67 proliferation index in *RASSF1A* WT and KO mice in normal‐appearing liver tissue, PREs, and tumors in *PIK3CA* H1047R‐injected mice; *n*(mice, KO) = 24; *n*(mice, WT) = 8. Visualization as estimated mean with 95% confidence interval. A mixed linear model was used. (E) Gross photograph of liver tumor spontaneously originating in an untreated RASSF1A KO mouse at 18‐month experimental time point (corresponding to mouse age of 20 months, left panel). Scale bar: 0.5 cm. Exemplary histological images and immunohistochemical characterization of well‐differentiated HCC transitioning into a lipid‐rich central tumor component in a *RASSF1A* KO PBS‐injected control mouse at the experimental time point of 12 months (right panels). Scale bars: 200 µm (large panel), 50 µm (inset), and 100 µm (smaller panels). T, tumor.

Next, we searched for potential histological differences between *RASSF1A* WT and KO mice. Histological analysis revealed that proliferative clusters, pericentrally located PREs, and tumors were characterized by a lipid‐rich phenotype and possibly shared the common ancestor of disseminated lipid‐rich cells, which can be observed in the liver acinus zone 3, corresponding to singular transfected cells. *PIK3CA* H1047R‐injected mice preferentially developed discontinuous but expansive PREs in the sense of confluent clusters, while *PIK3CA* E545K‐injected mice developed focal, coherent, and more rounded PREs. Tumors were predominantly well‐differentiated and displayed a relatively low nuclear‐cytoplasmic ratio and a low degree of nuclear pleomorphism while maintaining their lipid‐rich phenotype. Visual examination of Ki67 revealed an increase in proliferation in tumors of E545K‐injected mice, which was not readily apparent for the observed lipid‐rich clusters in the H1047R injection group. There was no histologically evident difference in the morphology of PREs and tumors between *RASSF1A* WT and KO mice (Fig. 2A,B).

To ascertain the preneoplastic nature of the described lesions, we conducted a deep learning‐based analysis using the software DeePathology™ STUDIO after training the recognition of Ki67‐positive and Ki67‐negative hepatocyte nuclei and the omission of background cells (such as lymphocytes and Kupffer cells) on manually selected regions of interest (Fig. S1). A mean amount of 5543 cells for *PIK3CA* H1047R‐injected mice and 1650 cells for *PIK3CA* E545K‐injected mice were analyzed per animal.

Mixed linear models (Fig. 2C,D) demonstrated that PREs and tumors have a significant increase in proliferation compared with normal‐appearing tissue for *RASSF1A* WT and KO in *PIK3CA* E545K‐injected mice. At the same time, there was no significant difference in proliferation between PREs and tumors for *RASSF1A* WT and KO in *PIK3CA* E545K‐injected mice. Also, the proliferation was not significantly different between *RASSF1A* WT and KO in *PIK3CA* E545K‐injected mice in normal‐appearing liver tissue and preneoplastic tissue (*P*‐values > 0.05). However, when comparing the proliferation in tumors between *RASSF1A* WT and KO in *PIK3CA* E545K‐injected mice, a statistically significant lower proliferation rate could be detected in *RASSF1A* KO mice (*P *= 0.0280).

For *PIK3CA* H1047R‐injected mice, a significant increase in proliferation of PREs compared to normal‐appearing liver tissue could only be found for *RASSF1A* KO. In *PIK3CA* H1047R‐injected mice, tumors had a significantly higher proliferation than PREs. Moreover, for these mice, a significant difference could be found by comparing *RASSF1A* WT and KO in PREs, with the restriction that the numbers of animals analyzed for these subgroups are small.

Finally, the two tumors spontaneously arising in *RASSF1A* KO mice in the combined control group were histologically examined. These tumors were extremely well‐differentiated, pure HCC with a low proliferation rate demonstrated by Ki67 immunohistochemistry. Interestingly, one of these tumors featured a lipid‐rich component with focally increased proliferation. This component displayed a hinted upregulation of stearoyl‐CoA desaturase‐1 (SCD1). In contrast, phosphorylated/activated phosphorylated extracellular signal‐regulated kinase (pERK) and phosphorylated RAC‐alpha serine/threonine‐protein kinase (pAKT) did not show an increased immunoreactivity (Fig. 2E).

Altogether, the low‐grade histology following a long latency confirms the limited oncogenic potential of *RASSF1A* inactivation alone. Proliferation showed a statistically significant tendency to be lower in *PIK3CA* E545K mutant tumors in *RASSF1A* KO mice than *RASSF1A* WT mice (which was not detectable in *PIK3CA* H1047R‐injected mice). Thus, irrespective of *RASSF1A*, we could define the multistep nature of PIK3CA mutant forms induced hepatocarcinogenesis.

### 3.2 *PIK3CA*‐induced tumors display a strong upregulation of canonical effectors

**Fig. 3:**
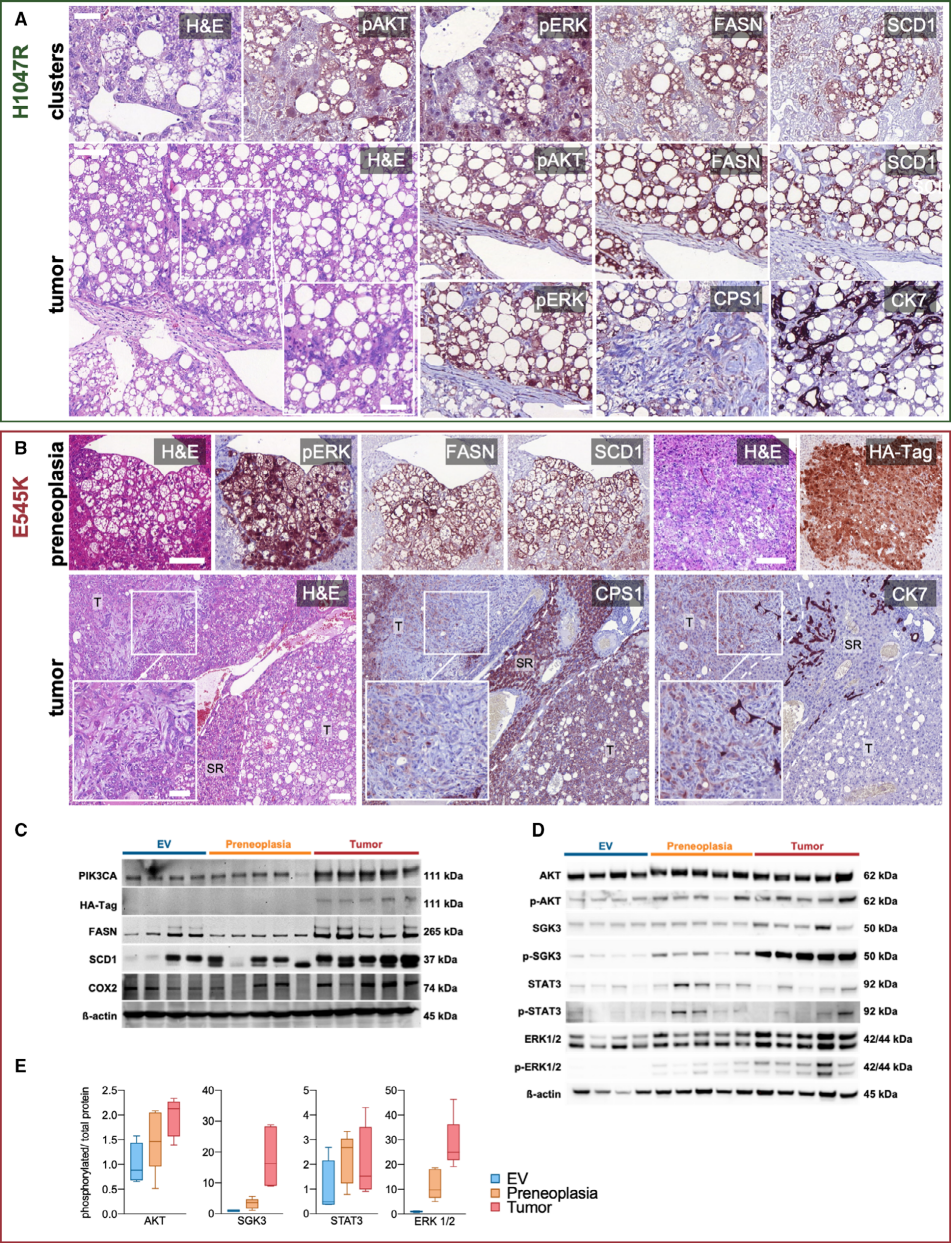
Activation of PIK3CA downstream effectors and the lipogenic phenotype in induced tumors. (A) Representative immunohistochemical analyses of preneoplastic clusters in one animal (upper row) and a tumor in one animal (lower row) generated by hydrodynamic tail vein injection of *PIK3CA* H1047R into WT mice. FASN and SCD1 represent master regulators of lipogenesis, while pAKT and pERK comprise downstream effectors of activated PIK3CA. Magnification inset highlighting focal cholangiocellular differentiation with the corresponding loss of CPS1 and positive staining for CK7 in one animal. Scale bars: 50 µm (except for magnification inset: 100 µm). (B) Representative immunohistochemical analyses of a preneoplastic lesion (upper row) and a tumor (lower row) generated by hydrodynamic tail vein injection of *PIK3CA* E545K into WT mice. Images from two different mice. Tumor magnification inset illustrates spindle cell morphology with combined weak expression of CPS1 and CK7. T, tumor. Scale bars: first row and second row large panel 100 µm, magnification inset 50 µm. (C) Western blot analysis shows effective upregulation of PIK3CA, HA‐tagged PIK3CA, FASN, SCD1, and pro‐inflammatory COX2 with increasing intensity in liver tissue containing preneoplastic lesions (*n* = 5 mice) and tumors (*n* = 5 mice) induced by *PIK3CA* E545K injections as compared to empty vehicle (EV) injections (*n* = 4 mice). (D) Western blot of PIK3CA downstream effectors pAKT, p‐SGK3, pERK1/2, and p‐STAT3 in liver lysates with preneoplastic lesions and tumors induced by *PIK3CA* E545K injections and liver lysates from EV injections with corresponding total protein levels of AKT, SGK3, ERK1/2, and STAT3. Loading control: β‐actin. Molecular weights of observed bands are marked on the right. (E) Phosphorylated protein/total protein ratios calculated from adjusted band intensities of the above western blot displayed as Tukey method box‐and‐whisker plots. (p)AKT, (phosphorylated) AKT; (p‐)SGK3, (phosphorylated) SGK3; (p‐)STAT3, (phosphorylated) STAT3.

Due to the absence of overt differences in tumorigenesis between *RASSF1A* WT and KO mice, we focused on *RASSF1A* WT mice. To confirm the hepatocellular nature and the activity of PIK3CA effectors in the PREs and tumors developed in PIK3CA mutant mice, we performed immunohistochemical analyses. Immunohistochemistry demonstrated that pAKT, the primary downstream target of PIK3CA, was strongly upregulated in preneoplastic clusters and tumors. Similarly, pERK was markedly induced, implying the activation of the ERK‐MAPK signaling pathway. Moreover, the master regulators of lipogenesis, SCD1 and fatty acid synthase (FASN), were strongly positive in E545K‐ and H1047R‐injected cohorts. Intense positivity for carbamoyl phosphate synthetase I (CPS1) protein, a highly specific hepatocyte marker, confirmed the hepatocellular nature of the neoplastic lesions. Compellingly, foci with cholangiocellular differentiation developed in PIK3CA E545K and H1047R mouse livers, as indicated by cytokeratin 7 (CK7) immunoreactivity. An overlap of CK7 with CPS1 was detected, which agrees with the hypothesis that the cholangiocellular components originate from transdifferentiation rather than from separate cholangiocarcinogenesis (Fig. 3A,B) [52]. At the protein level, we verified the gradual upregulation of PIK3CA, pAKT, p‐serum/glucocorticoid‐regulated kinase family member 3 (SGK3), pERK1/2, p‐signal transducer and activator of transcription 3 (STAT3), FASN, and SCD1 in PREs and tumors in PIK3CA E545K‐injected mice. The activation of the aforementioned phosphorylated proteins was also evident in terms of an increased ratio over the total amount of the respective proteins. Notably, prostaglandin‐endoperoxide synthase 2 (COX2), known to induce tumor‐promoting inflammation through increased prostaglandin E2 synthesis [53], was also upregulated. Recently, eicosanoid metabolism has been increasingly recognized as an essential mechanism of PIK3CA‐mediated oncogenicity (Fig. 3C–E) [54]. When looking into COX2 expression on an immunohistochemical level, enhanced immunoreactivity could be detected preferentially in tumor vasculature, while an upregulation in neoplastic cells was only hinted (Fig. S2). Interestingly, overexpression of COX2, which produces prostaglandin E2, an important angiogenic factor, in tumor‐associated blood vessels, has been previously described in human HCC [55].

Next, we carried out transmission electron microscopy to evaluate the tumors on an ultrastructural level. In concordance with the histologically visible empty vacuoles, tumor cells showed abundant intracytoplasmic microvesicular lipid droplets. These differed markedly from an example of spontaneous liver steatosis in a control mouse, where only a few scattered, larger intracytoplasmic lipid droplets could be found. The extensive cytoplasmic accumulation of lipid vesicles underlines the profound reliance of these tumors on lipogenesis (Fig. S3A). Subsequent Sudan IV histochemical staining on fresh frozen tissue confirmed the extensive presence of intracytoplasmic triglycerides in the tumors (Fig. S3B).

To assess the potential involvement of fibrosis and stellate cells on hepatocarcinogenesis in our model, we performed Picro Sirius Red histochemical stainings on untreated mice and mice harboring PREs or tumors. These stainings did not show an increase in fibrosis along the process of hepatocarcinogenesis. Moreover, to test for a potential activation of stellate cells, desmin and alpha‐smooth actin immunohistochemistry was carried out, which did not demonstrate an increase in the number or activation status of stellate cells in the neoplastic lesions (Fig. S4A). In concordance with these results, electron microscopy did not show increased or activated stellate cells when comparing tumors and normal liver tissue (Fig. S4B).

Overall, we observed a marked upregulation of PIK3CA canonical downstream effectors. Massive lipogenesis, paralleled by robust upregulation of FASN and SCD1 lipogenic enzymes, was one of the earliest events in PIK3CA‐mediated hepatocarcinogenesis.

### 3.3 Identification of additional putative targets in PIK3CA‐driven hepatocarcinogenesis

**Fig. 4:**
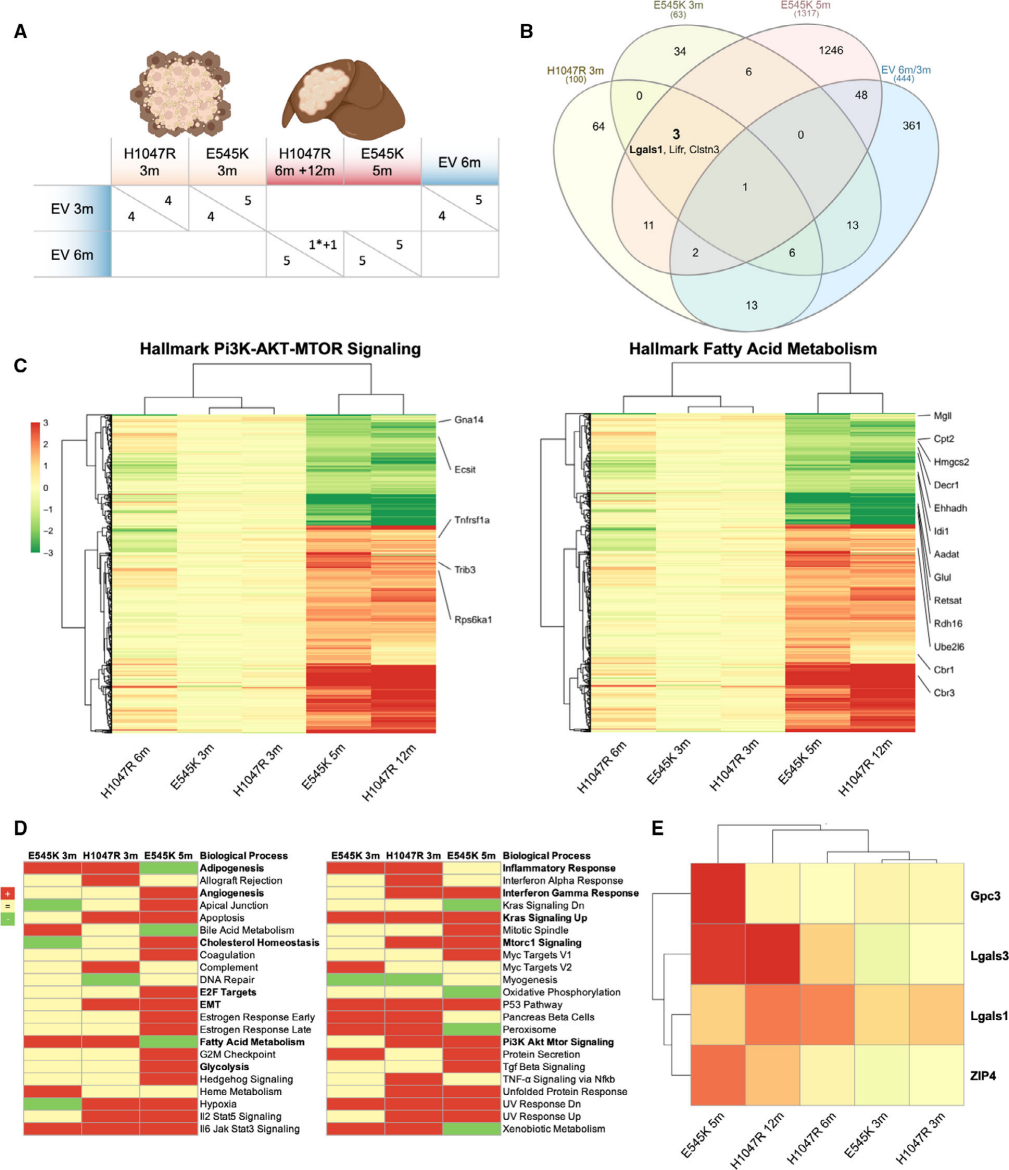
Metabolic signatures and identification of the novel effectors Gal‐1, Gal‐3, and ZIP4 in *PIK3CA*‐dependent hepatocarcinogenesis. (A) Overview of samples and comparison groups for cDNA microarray analysis. Preneoplastic lesions were compared to age‐matched controls injected with the EV. EV‐injected 3‐month time points were contrasted to EV‐injected 6 months to rule out age‐dependent effects. Only one tumor at 12 months was available from the H1047R injection arm. The asterisk indicates a *PIK3CA* H1047R‐induced confluent preneoplastic lesion analyzed at 6 months. (B) Venn diagram of cDNA microarrays exhibiting overlap of regulated genes in the prespecified comparison groups. Selection criteria were Log_1.5_ change and *P* < 0.01 for H1047R 3 months, E545K 3 months and control 6 versus 3 months, Log_2_ fold change and adj *P* < 0.05 for E545K 5 months. The numbers of overlapping and uniquely altered genes are given. The three genes conjointly called in the *PIK3CA* injection groups are mentioned, while *Lgals1* encoding for Gal‐1 is written in bold. (C) Heatmap of differentially expressed genes with selection criteria |log_2_FC| > 1, adj. *P* < 0.05 and counts > 3 as determined in the E545K 5 months of comparison group. Specified genes are comprised in the GSEA hallmark gene sets PI3K‐AKT‐MTOR signaling (left panel) and fatty acid metabolism (right panel). Gradient scale color codes for Log_2_ fold change. A cluster dendrogram is shown on the left and above. (D) GSEA was performed to identify significantly upregulated (red) or downregulated (green) biological processes in *PIK3CA* E545K 3 months, H1047R 3 months, and E545K 5 months of injection groups. Particularly relevant processes implicated with hepatocarcinogenesis are reported in bold. (E) Target genes *glypican‐3* (*Gpc3*), *Lgals‐3* (*Gal‐3*), and *ZIP4* fulfilled the selection criteria prementioned above in (B) and (C). *LGALS1* (*Gal‐1*) met the selection criteria described in (B). *Gpc3* serves as an internal positive control as upregulation is expected in HCC. Target genes were manually chosen based on novelty, potential pharmacological targetability (*Gal‐1* and *Gal‐3*), and early upregulation in preneoplastic lesions (*Gal‐1*). Presentation as a heatmap with identical color‐coding as in Panel (C).

Although lipogenesis is an established mediator of hepatocarcinogenesis, it remains challenging to target it selectively without significant systemic adverse effects due to its ubiquitous involvement in metabolism [56]. Therefore, the search needs to be widened to identify novel targets that can be pharmacologically manipulated. Thus, we performed gene expression microarray analyses of *PIK3CA* H1047R‐ and E545K‐induced PREs and tumors (Fig. 4A). *Lgals1*, which encodes Gal‐1, was among the few genes concomitantly upregulated in *PIK3CA*‐induced PREs and tumors compared with age‐matched livers from EV‐injected mice. We reached this conclusion based on the depicted Venn diagram [57] (Fig. 4B). This finding was intriguing for several reasons. Gal‐1, being a 14 kDa beta‐galactose‐specific binding protein [58], is known to be increased in numerous neoplasms, including primary hepatic tumors [59]. Moreover, Gal‐1 is an adverse prognostic marker in HCC [60, 61]. And finally, Gal‐1 has been linked to the PI3K‐AKT‐mTOR signaling, thereby enhancing in HCC cell lines [62]. Given that Gal‐1 expression was already elevated in preneoplastic tissue and retained during tumor progression, it was selected for further investigation.

In accordance with immunohistochemical results, induction of the PI3K‐AKT‐mTOR pathway was detected by the Hallmark Gene Set Enrichment Analyses (GSEA) [40]. The latter revealed an upregulation in PREs and/or tumors of pathways such as inflammatory response, interferon‐gamma response, angiogenesis, cholesterol homeostasis, E2F targets, EMT, KRAS signaling, and Myc targets. Surprisingly, fatty acid metabolism was exclusively upregulated in PREs, suggesting a transition in the direction of increased independence from lipid metabolism in the later stages of carcinogenesis. In addition, several genes in the Hallmark PI3K‐AKT‐mTOR gene set (upregulation of Tnfrsf1a, Trib3, and Rps6ka1; downregulation of Gna14, and Ecsit) and the Hallmark Fatty Acid Metabolism gene set (upregulation of Cbr3, Cbr1, Ube2l6; downregulation of Rdh16, Retsat, Glul, Aadat, Idi1, Ehhadh, Decr1, Hmgcs2, Cpt2, and Mgll) met the following criteria |log_2_FC| > 1, adj. *P* ≤ 0.05 and counts > 3 as determined in the E545K 5 months of comparison group (Fig. 4C,D).

Further analysis of the microarray data for targetable proteins with a cutoff of Log2 fold change and adj *P* < 0.05 in the PIK3CA E545K‐injected group revealed *Lgals‐3* and *ZIP4* as additional compelling targets (Fig. 4E). Lgals‐3 encodes galectin‐3 (Gal‐3), another member of the lectin family, implicated in the development of liver cirrhosis [63], immunosuppression in cancer [64], and with a negative prognostic value in HCC [65]. Zrt‐Irt‐like protein 4 (ZIP4) is a cellular zinc transporter [66, 67], upregulated in HCC, where it represses apoptosis and increases migration [68]. Also, ZIP4 is correlated with poor survival in HCC patients [69].

### 3.4 Galectin‐1, ZIP4, and galectin‐3 are highly expressed in PIK3CA‐induced lesions

**Fig. 5:**
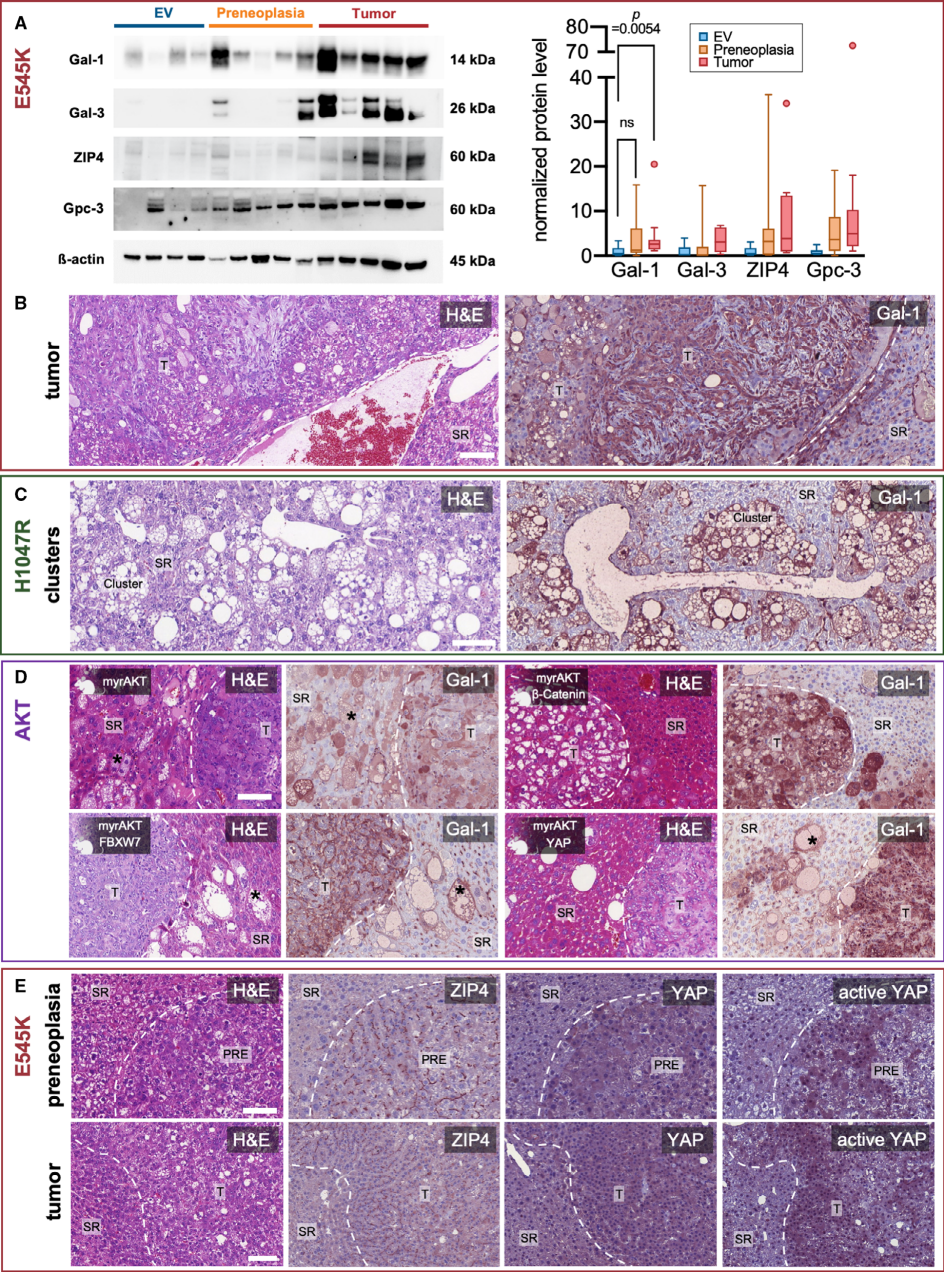
Validation of target proteins and early upregulation of galectin‐1 in precancerous lesions. (A) Western blot analysis demonstrating increased band intensities for Gal‐1, Gal‐3, ZIP4, as well as glypican‐3 (Gpc3) in tumorous and in part also in precancerous liver tissue (left panel) in *PIK3CA* E545K‐injected mice, as compared to empty vehicle injections. β‐actin was used as a loading control. Molecular weights of observed bands are marked on the right. Western blot quantification of band intensities normalized on corresponding β‐actin signals (right panel) yielded a significant increase in Gal‐1 intensity of tumors versus preneoplastic lesions (*N* = 3 western blot repeats). Kruskal–Wallis test was used. Tukey method box‐and‐whisker plots are displayed (ns = not significant; *P* > 0.05). Quantification of two western blot repeats each is shown adjacently for the remaining target proteins. (B) Gal‐1 immunohistochemistry of a *PIK3CA* E545K injection‐induced tumor with spindle cell and steatotic components shown in one mouse, representative for a total 10 stained mice. Scale bar: 100 µm. (C) Gal‐1 immunohistochemistry of preneoplastic pericentral clusters resulting from *PIK3CA* H1047R injection. Representative for a total of 5 mice stained. Scale bar: 100 µm. (D) Gal‐1 overexpression in various mouse models generated by hydrodynamic tail vein injections of either activated *AKT* with myristoylation sequence alone or in conjunction with *β‐Catenin*, *FBXW7*, and *YAP*. One animal in each injection group was exemplarily stained and is shown here. Asterisks (*) point out preneoplastic single cells. Scale bar: 100 µm. (E) ZIP4, YAP, and active YAP immunohistochemistry of a preneoplastic lesion (upper row) and a tumor (lower row) developing in a *PIK3CA* E545K‐injected mouse. Note the increased membranous expression of ZIP4 in contrast to surrounding liver tissue. A total of four mice were stained with visually similar results. Scale bars: 100 µm. PRE, preneoplasia; T, tumor.

To validate the gene expression microarray analysis results, the expression of Gal‐1, ZIP4, and Gal‐3 was investigated by western blot analysis. All proteins were strongly upregulated in *PIK3CA* E545K‐induced tumors, and it was statistically significant for Gal‐1. Moreover, a tendency of upregulation occurred in PREs (Fig. 5A). To corroborate these findings, we performed immunohistochemistry. Gal‐1 appeared robustly increased in tumors and PREs from *PIK3CA* mutant‐injected mice (Fig. 5B,C).

To investigate a potential link with the PIK3CA‐AKT‐mTOR pathway, we additionally examined different mouse models that were induced by the injection of myristoylated *AKT* either alone [70] or in combination with other genes, such as *β‐catenin* [71], *F‐box and WD repeat domain‐containing 7* (*FBXW7*) [72], and *YAP* [73]. Notably, the latter two combinations induce cholangiocarcinogenesis [73]. In all mouse models, tumors exhibited strong immunoreactivity for Gal‐1 compared to the surrounding liver tissue. Remarkably, Gal‐1 positivity was also evident in isolated lipid‐rich preneoplastic hepatocytes (Fig. 5D).

Furthermore, we conducted immunohistochemical analyses of ZIP4 protein expression. Pronounced membranous immunolabeling for ZIP4 was detected in tumors and PREs of *PIK3CA* E545K‐injected mice. Interestingly, there was also a corresponding upregulation of the Hippo pathway effector YAP and active YAP (Fig. 5E).

### 3.5 The galectin inhibitor OTX008 synergizes with PI3K inhibitors *in vitro*

In light of Gal‐1 overexpression in PIK3CA‐AKT‐dependent tumors, we assessed the importance of Gal‐1 on HCC cell growth *in vitro*. Thus, we treated the PLC/PRF/5 and HLE HCC cell lines with the PI3K inhibitor alpelisib, either alone or associated with the Gal‐1 inhibitor OTX008. Alpelisib has proven its clinical actionability in the large phase 3 trial SOLAR‐1, which resulted in the drug's approval by the Food and Drug Administration for hormone receptor‐positive, human epidermal growth factor receptor 2–negative breast cancer [74]. In contrast, OTX008, a calixarene derivative designed to bind the Gal‐1 β‐sheet conformation [75], has only undergone early clinical testing in the form of a dose‐finding phase 1 study in patients with advanced solid tumors [76].

**Fig. 6:**
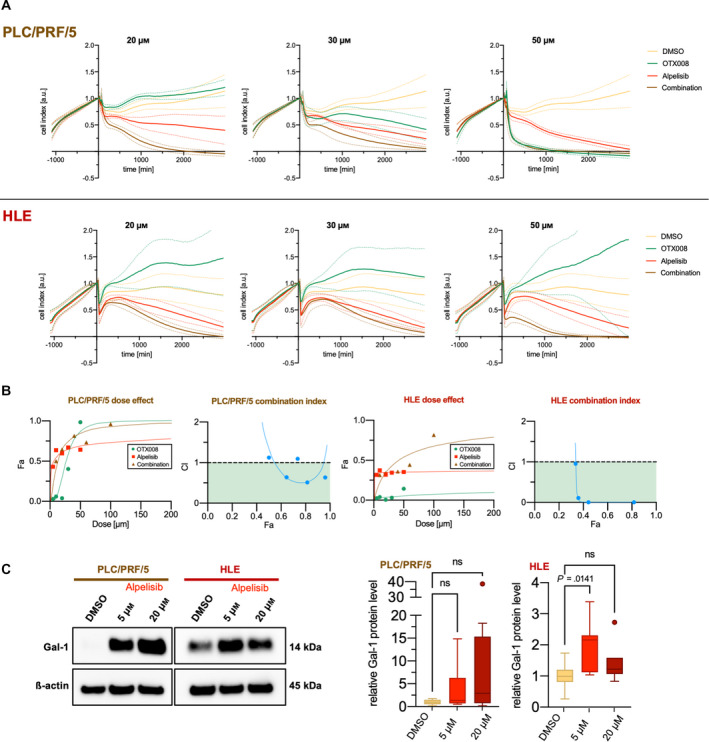
Strong synergism of Gal‐1 inhibitor OTX008 and PIK3CA inhibitor alpelisib in human HCC cell lines. (A) Time‐dependent proliferation and cytotoxicity profiles generated by impedance measurements using an xCELLigence® device. PLC/PRF/5 cells are shown in the upper row, HLE in the lower row. Addition of matched DMSO, OTX008, alpelisib at timepoint 0 in the outlined concentration either as a single or combined treatment after initial outgrowth for ~ 24 h. The cell index was normalized to the timepoint of drug addition. Solid lines signify mean and dashed lines 95% confidence interval. The illustrated data consist of three experimental repeats with 2–3 replicates acquired for each cell line and concentration. (B) CompuSyn®‐software calculation results to determine drug synergism of OTX008 and alpelisib in PLC/PRF/5 (two left panels) and HLE (two right panels). Values obtained by xCELLigence® cell viability analysis at 24 h were employed, and the results of one representative experiment are shown. Dose–effect curves (Fa = fraction affected), and corresponding combination index plots are provided. A combination index (CI) < 1 is interpreted as synergism. CI values of < 1 (colored in green), 1, > 1 can be interpreted as synergistic, additive, and antagonistic effects, respectively. (C) Exemplary western blot illustrating a tendency of increased Gal‐1 levels in response to alpelisib treatments in the HCC cell lines PLC/PRF/5 and HLE (left panel). A quantification of three treatment repeats with 2–3 replicates each was undertaken (right panel). Kruskal–Wallis test was used; ns, not significant.

Combining the two drugs led to significantly (by evaluation of 95% confidence intervals) [77] higher antigrowth properties than either of the two drugs alone. This effect was evident for combined concentrations of 20 and 30 µm in PLC/PRF/5 and 20 and 50 µm in HLE cell lines. Notably, OTX008 only displayed relevant antigrowth effects as a single drug in PLC/PRF/5 at the examined concentrations up to 50 µm (Fig. 6A). To confirm the synergistic effect, the combination index was calculated based on the median‐effect principle of the mass‐action law [78]. A combination index < 1 signifying synergism was attained for most combined concentrations in both PLC/PRF/5 and HLE cells (Fig. 6B).

To determine whether alpelisib treatment influences Gal‐1 expression, we treated the two cell lines with 5 and 20 µm, respectively. Western blot analyses indicated an upregulation of Gal‐1 in response to alpelisib treatment after 48 h. However, this trend was only statistically significant (*P* = 0.0141) in the quantification at an alpelisib concentration of 5 µm in the HLE cell line (Fig. 6C). Although this finding contrasted our expectations, it might still explain the synergistic effects observed for combination treatments with OTX008, given that previous studies inferred a correlation between the antiproliferative effect of OTX008 and Gal‐1 expression [79].

To study the observed synergy in a mutation‐specific background, the SNU387 and SNU449 HCC cell lines were lentivirally transduced with *PIK3CA* E545K, *PIK3CA* H1047R, and EGFP plasmids. The yielded stably transfected cell lines were functionally characterized using western blots. Increased levels of PIK3CA, phosphorylated AKT at Ser473 and Thr308 residues, and phosphorylated ERK1/2 (Thr202/Tyr204) confirmed the activation of the PI3K pathway (Fig. S5). The IC50 values of the stably transfected HCC cell lines were calculated using the crystal violet cytotoxicity assay after applying incremental concentrations of either alpelisib or OTX008. Transfection with the two *PIK3CA* mutant forms markedly decreased alpelisib IC50 values compared to *EGFP‐*transfected cell lines, while a reduction of OTX008 IC50 values based on the transfected constructs was not apparent (Fig. S6). Finally, to evaluate the combined cytotoxicity of alpelisib and OTX008 based on the *PIK3CA* transfection status, incremental concentrations of the two drugs were applied either alone or in combination, and combination indices were calculated based on the Chou‐Talalay method. A trend toward a higher synergy (i.e., CI < 1) could be observed in all of the *PIK3CA* stably transfected cell lines, with the most pronounced effects for SNU387. Notably, *EGFP*‐transfected cells even displayed an antagonism when combining the two drugs, while in *PIK3CA* E545K transfected cells, a high synergy could be observed for all tested concentrations (Fig. S7).

To evaluate the generalizability of the observed synergy with OTX008 to other PI3K inhibitors, treatments of PLC/PRF/5 cells with 1 µm taselisib, 1 µm buparlisib, or 1 µm alpelisib with or without the addition of 20 µm OTX008 were conducted for 72 h. The mean excess over Bliss score was 0.1685 for alpelisib, 0.1164 for buparlisib, and 0.1548 for taselisib in three internal replicates, indicating a similarly strong synergistic effect for all three of these compounds (Fig. S8).

Moreover, as a discovery approach for more potential interaction partners of OTX008 (at a concentration of 20 µm), we conducted a compound library screening including 315 approved drugs (at a concentration of 1 µm). All four PI3K inhibitors included in the screening demonstrated a synergistic effect in combination with OTX008. The excess over Bliss score was 0.0931 for Duvelisib, 0.0924 for Copanlisib, 0.0723 for Idelalisib, and 0.0189 for buparlisib (Table S2). Using the threshold of viability from combination treatment < 0.6 and *Z* score of EOB > 1.5, several more putative synergistic interaction partners of OTX008 could be identified (Fig. S9 and Table S1). Pacritinib, a Janus‐Kinase 2‐inhibitor, emerged as the compound with the highest synergistic effect. On top of that, several other tyrosine kinase inhibitors, such as Axitinib, Apatinib, Crizotinib, Sorafenib, Crenolanib, and Nintedanib, and the two EGFR inhibitors Neratinib and Afatinib also reached the defined threshold. Thus, in advanced clinical trials, several approved drugs could be considered worthwhile candidates for combination treatments with OTX008 and warrant further investigations.

### 3.6 Differential effectors of *PIK3CA* E545K and *PIK3CA* H1047R

In an attempt to shed light on the observed difference in oncogenicity and tumor latency between *PIK3CA* E545K and H1047R, we performed comparative gene expression microarray analyses (using the Applied Biosystems™ GeneChip™ Clariom S human array) of the stably transfected SNU387 and SNU449 HCC cell lines. Among the significantly upregulated genes in comparing the two mutant forms against *EGFP* and WT, *PIK3CA* could be detected in both cases, serving as an internal positive control. Moreover, vascular cell adhesion molecule 1, encoding for a protein connected to angiogenesis and metastasis [80], was strongly downregulated on a single gene level in H1047R compared with WT/*EGFP* and E545K. An overview of all significantly regulated genes is provided in Figs S10 and S11. To search for potentially different effector pathways, we performed GSEA analyses based on the Panther pathway gene set [81] (Fig. S12). Commonly upregulated pathways for the two *PIK3CA* mutant forms included the following: apoptosis signaling pathway, hypoxia response via HIF activation, and insulin/IGF pathway‐protein kinase B signaling cascade. Differentially activated pathways comprised the PI3 kinase pathway (which by this method was significantly upregulated only in H1047R cells), the interleukin signaling pathway, chemokine and cytokine signaling, and the plasminogen activating cascade, which were upregulated in H1047R cells. In contrast, in E545K cells, the Toll receptor signaling pathway, CCKR signaling, angiogenesis, and VEGF cascade were induced. The only significant hit (false discovery rate > 0.05) for the direct comparison of the two *PIK3CA* mutant forms was the plasminogen activating cascade, with increased activation in H1047R cells.

To summarize, *PIK3CA* H1047R preferentially induced inflammatory pathways (interleukin and chemokine signaling), while *PIK3CA* E545K instead promoted angiogenic pathways (angiogenesis and VEGF signaling). Albeit no distant metastases were detected in our mouse model, microarray analyses in human HCC cell lines hint at a ‘neovascular/metastatic phenotype’ for E545K and an inflammatory phenotype for H1047R.

### 3.7 An interdependence with SCD1 links galectin‐1 to lipogenesis

**Fig. 7:**
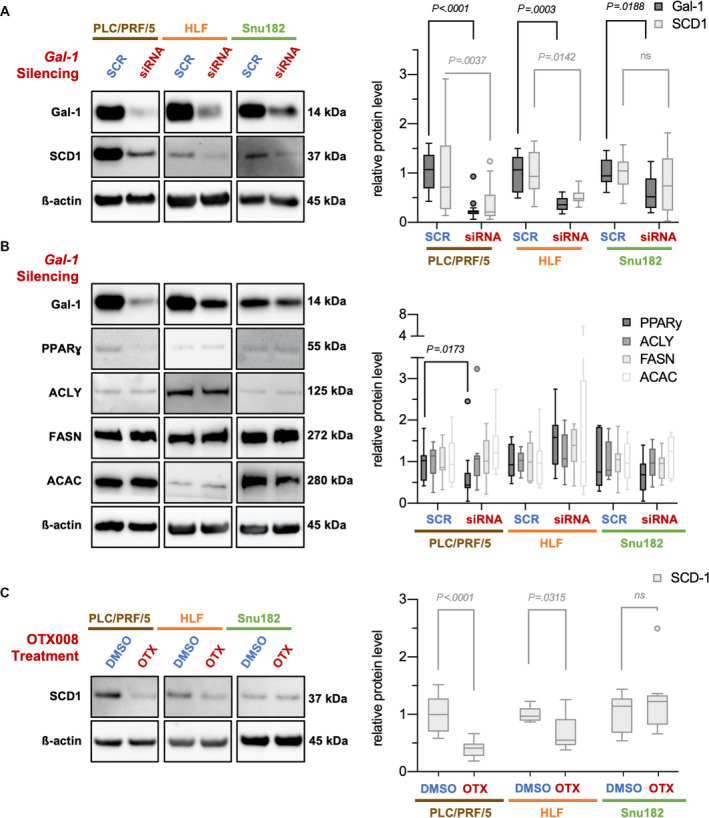
Interdependence of Gal‐1 and SCD1 expression in human HCC cell lines suggests regulation of lipid synthesis. (A) siRNA‐mediated *Gal‐1* silencing elicited a decrease in SCD1 protein levels in the human HCC cell lines PLC/PRF/5, HLF, and Snu182 as exemplified in western blot analyses (left panel). Molecular weights of observed bands are noted. Quantification of yielded bands was performed (right panel). (B) In contrast, siRNA‐mediated *Gal‐1* silencing did not yield a regulation of other important lipogenic enzymes, such as ACLY, FASN, and ACAC. At the same time, a tendency for proliferator‐activated receptor gamma (PPARɣ) reduction could be observed in PLC and Snu182 cell lines. *n*(PLC/PRF/5) = 3 cell culture experimental repeats with 3 cell culture replicates + 1 cell culture experimental repeat with 6 cell culture replicates. *n*(HLF, Snu182) = 3 cell culture experimental repeats with 3 cell culture replicates. (C) 20 µm OTX008 treatment for 24 h likewise led to a decrease in SCD1 in PLC/PRF/5 and HLF cell lines as demonstrated by Western blot (left panel). Molecular weights of observed bands are noted. Quantification of yielded bands was performed (right panel). Intensities were normalized to the mean of control for all tested proteins. Tukey method box‐and‐whisker plots are displayed (right panels). Mann–Whitney tests were employed; ns, not significant. All pairwise comparisons in the right panel in (B) were not significant, except for the one displayed.

In the search for a potential mechanism of cytotoxicity, we assessed whether Gal‐1 is associated with lipogenesis in HCC. A functional connection between Gal‐1 and the expression of lipogenic factors has recently been elucidated in livers of *Gal‐1* KO mice, where FASN, acetyl‐CoA carboxylase 1 (ACAC), and SCD1 were downregulated in animals fed a high‐fat diet [82]. For this purpose, we performed western blot analysis of SCD1 expression following Gal‐1 siRNA‐mediated silencing (Fig. 7A). As expected, Gal‐1 levels were downregulated in all tested HCC cell lines (*P*‐values < 0.05). Furthermore, decreased SCD1 levels in PLC/PRF/5, HLF, and Snu182 HCC cell lines subjected to Gal‐1 knockdown were observed. Quantitative analysis of band intensities supported this observation in PLC/PRF/5 and HLF and reached statistical significance (*P*‐values < 0.05), which was, however, not met in Snu182 (*P* = 0.34). In addition, we tested the effect of Gal‐1 downregulation on other lipogenic enzymes, including peroxisome proliferator‐activated receptor gamma (PPARγ), ATP citrate synthase (ACLY), FASN, and ACAC. Among these, only PPARγ showed a statistically significant downregulation (*P* = 0.0173) upon Gal‐1 silencing in the PLC cell line (Fig. 7B).

As a further validation for the observed effect on SCD1 protein levels, OTX008 treatments were performed to inhibit Gal‐1 functionally. Similar to the Gal‐1 silencing data, a downregulation of SCD1 protein levels was evident and statistically significant for PLC/PRF/5 (*P* < 0.0001) and HLF (*P* = 0.0315) cell lines. In contrast, no significant effect could be observed for the Snu182 cell line (Fig. 7C).

Taken together, we could deduce a dependence of SCD1, one of the master regulators of lipogenesis, on Gal‐1 expression in HCC.

## 4 Discussion

Both the PI3K‐AKT‐mTOR and the Ras‐MAPK pathways have been commonly implicated in hepatocarcinogenesis [83, 84]. This finding raised the question of possible interdependence and cooperativity between these signaling networks. RASSF1A as an inhibitor of Ras activity with a high methylation frequency in HCC [13] represented a promising candidate as an oncogenic signaling node, mediating the two pathways' effects. We addressed the question of RASSF1A oncogenicity using a murine *RASSF1A* KO model with hydrodynamic tail vein injection of *PIK3CA* H1047R and E545K mutant forms. Surprisingly, we found that *PIK3CA* mutant forms elicited hepatocarcinogenesis irrespective of *RASSF1A* status, precluding hypothesized cooperativity.

Another unanticipated conclusion from these experiments was that *PIK3CA* mutant forms H1047R and E545K alone could induce hepatocarcinogenesis. Previous experiments suggested that combination partners such as activated NRAS or c‐Met are necessary for PIK3CA to drive hepatocarcinogenesis [35]. Notably, *PIK3CA* E545K exhibited a much stronger oncogenic potential, giving rise to HCCs in most mice as early as 3 months after injection. *PIK3CA* H1047R, in contrast, only produced sporadic tumors after 12 months, which is opposite to findings in breast cancer [85]. The generation of this novel purely *PIK3CA*‐induced mouse model opens the door to further investigations into the PI3K‐AKT‐mTOR pathway in hepatocarcinogenesis. The availability of such a model is of high interest. First, because 4–6% of human HCCs harbor a PIK3CA mutation [7, 33], which could be amenable to targeted therapies. Second, the PI3K‐AKT‐mTOR pathway activation often occurs in HCCs regardless of the PIK3CA mutational status [32]. Importantly, we could establish *PIK3CA*‐mediated hepatocarcinogenesis as a multistep process including PREs, enabling the study of early predominant mechanisms. This finding paralleled observations that have been made in mice hydrodynamically injected with activated *AKT* [70]. When we examined the tumors induced in this model, we found an increase in the canonical effectors of PIK3CA, such as pAKT, pERK, p‐SGK3, and p‐STAT3.

In addition to these canonical targets, we revealed a pronounced increase in lipogenesis in tumors and even PREs, which was evident on several experimental layers: an increase in the level of the master regulators of lipogenesis SCD1 and FASN, an upregulation of lipid metabolism by gene expression analysis, and a lipid‐rich phenotype to an extent, where tumor cells were packed with lipid droplets as seen by electron microscopy. *De novo* lipogenesis is a cancer hallmark. The synthesis of fatty acids provides tumor cells with metabolic intermediates essential for synthesizing the membrane lipid bilayer, energy storage in the form of triglycerides, and signaling molecules [56]. In a related murine HCC model, it has antecedently been demonstrated that the deletion of *FASN* abolishes *AKT*‐induced carcinogenesis [86]. Therefore, inhibitors of FASN and other lipogenic enzymes can represent enticing therapeutic options, was it not for dose‐limiting toxicity, which can arise in adipose and liver tissue [56]. Consequently, it is an important endeavor to widen the search for potential therapeutic targets that can influence lipogenesis.

Using gene expression analyses, we detected several candidates, which could serve as potential targets. Among them, we identified Gal‐1 as a protein already strongly upregulated in PREs, underlining its putative importance in hepatocarcinogenesis. A captivating finding was the interrelation of Gal‐1 expression and lipogenesis by regulating SCD1 protein levels. The incentive to analyze a potential link between Gal‐1 and lipogenesis came from a recent report showing the downregulation of FASN, ACC1, and SCD1 expression in adipose and hepatic tissues from *Gal‐1* KO mice [82]. This regulation could well play a critical role in hepatocarcinogenesis and warrants further experimental elucidation.

Intriguingly, Gal‐1 is accessible to pharmacological targeting, such as the calixarene derivative OTX008, already tested in an early clinical trial [76]. In this study, the drug was administered to HCC cell lines. While there were limited effects when OTX008 was applied alone, a strong synergism could be detected in combination with the PIK3CA inhibitor alpelisib. Indeed, previous reports directly put Gal‐1 in the context of PI3K‐signaling [87]. In our hands, alpelisib treatment increased Gal‐1 expression, which might sensitize cells to OTX008 treatment. In the phase 1 trial mentioned before, plasma concentrations ‘> 1 µm over 12 h after administration’ [76] were achieved. Given that the concentrations of OTX008 were comparatively high in our experiments (~ 20 µm), we must exert at least some caution in interpreting these findings. Of note, synergistic effects of OTX008 with either Sorafenib or Everolimus have been reported in HCC cell lines before [79], which further substantiates the interaction of Gal‐1 with the PI3K‐AKT‐mTOR pathway. In addition, multidrug pharmacologic screening revealed several more candidate drugs acting synergistically with OTX008, including JAK, EGFR and other tyrosine kinase inhibitors, highlighting its potential in combination therapies.

Finally, two additional upregulated targets identified in this study with potential therapeutic implications were Gal‐3 and ZIP4. Besides being repeatedly implicated in inflammation [88], Gal‐3 is also linked to lipid metabolism since FASN, proliferator‐activator receptor gamma, and fatty acid‐binding protein 4 were significantly reduced in Gal‐3 KO mice [89]. ZIP4 emerged as a highly interesting target protein since we found a correlating upregulation of YAP and active YAP. ZIP4 and YAP have recently been demonstrated to be commonly responsible for EMT control in pancreatic cancer [90]. Further research is required to verify the context of these proteins in PIK3CA‐mediated carcinogenesis and determine the effect of ZIP4 on EMT in HCC.

**Fig. 8:**
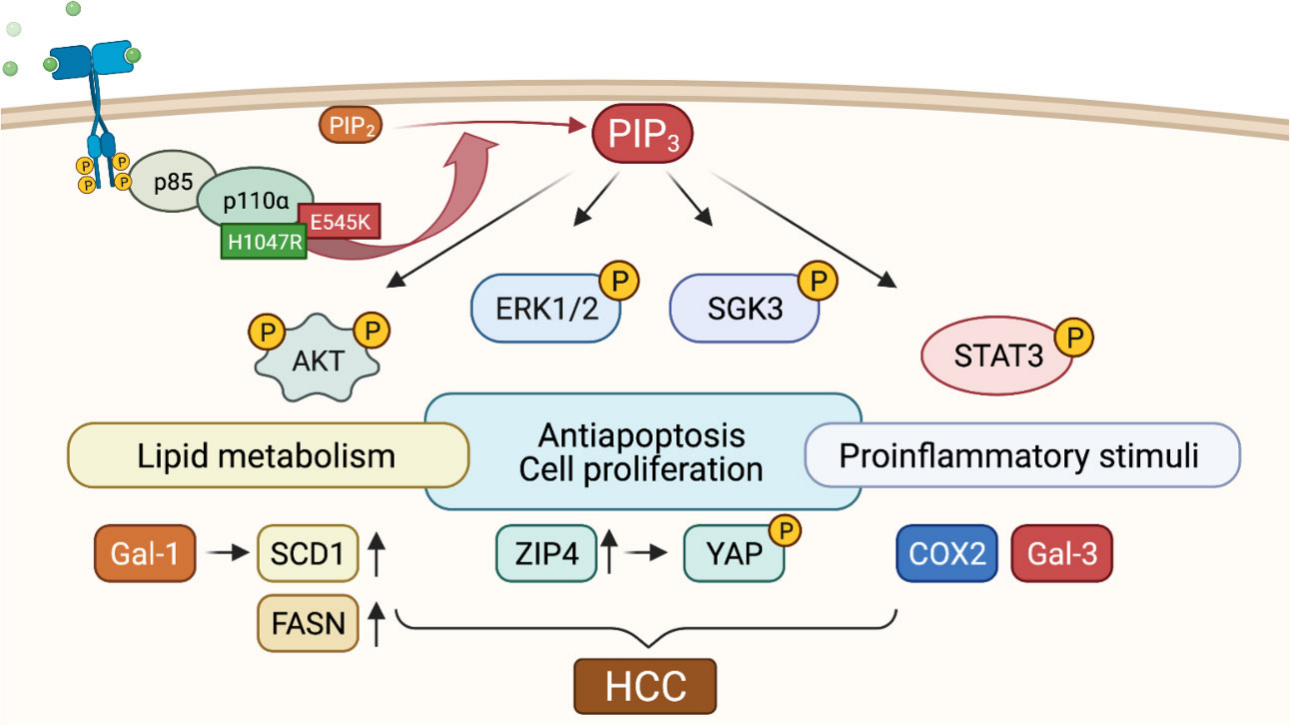
Graphical depiction of proposed PIK3CA‐induced hepatocarcinogenesis. H1047R and E545K mutations result in a receptor‐independent constitutive activation of PIK3CA. In complex with the regulatory subunit p85, the conversion of phosphatidylinositol (4,5)‐bisphosphate (PIP_2_) into PIP_3_ is catalyzed. PIP_3_ activates the downstream effectors AKT, ERK1/2, phosphorylated SGK3, and STAT3, either directly or via the mediation of phosphoinositide‐dependent protein kinase 1. AKT has a pivotal role in the pathway by inducing antiapoptosis and cell proliferation. Cellular metabolism is veered toward lipid biosynthesis by increased expression of the master lipid regulators SCD1 and FASN. Gal‐1 upregulation acts in unison by eliciting the upregulation of SCD1 and could account for an accessible therapeutic target. Furthermore, the elevation of ZIP4 encompasses YAP activation, thereby potentially providing a bridge to the Hippo signaling pathway. Finally, in correlation with the macroscopic steatohepatitic phenotype, pro‐inflammatory proteins such as COX2 and Gal‐3 are stimulated. Cooperatively, these alterations induce the formation of HCC.

Overall, the present study has determined the independence of *RASSF1A* KO‐mediated and *PIK3CA*‐driven hepatocarcinogenesis. We have established both *PIK3CA* stably transfected cell lines that reveal the targetability of oncogenic PIK3CA mutations and an HCC mouse model of high translational value concerning PI3K‐targeted therapies. Furthermore, this model allowed us to confirm the paradigm of *PIK3CA*‐driven carcinogenesis (in terms of canonical effectors of the pathway) and enhance our understanding of PIK3CA oncogenic properties. We ascertained the pivotal role of lipogenesis and discovered novel putative effectors, including Gal‐1, with therapeutic actionability (Fig. 8). These findings pave the way for ensuing investigations, such as searching for further combination partners of Gal‐1 and the study of *PIK3CA*‐dependent oncogenicity in *Gal‐1* KO mice.

## 5 Conclusion

*PIK3CA* E545K and H1047R mutant forms confer oncogenic potential irrespective of RASSF1A *in vivo*. Induced HCCs showed a concomitant upregulation of lipogenesis and Gal‐1 expression. The regulation of SCD1 by Gal‐1 and the synergism of alpelisib and OTX008 suggest Gal‐1 as a potential therapeutic target in HCC.

## Conflict of interest

The authors declare no conflict of interest.

## Author contributions

KE, KU, ME, AS, and DFC conceptualized the study; KU, KE, ME, AS, and DFC contributed to methodology; AS, KM, TI, KH, and AC provided software; KU, AS, and DFC validated the study; TI, AS, KU, KE, and AC involved in formal analysis; AS, KA, LR, KA, TI, AC, L‐M‐GP; SM‐R, HX, and GC investigated the study; ME, XC, FD, AT, and CB provided resources; AS, AC, and KM curated the data; AS and KU wrote—original draft preparation; AS and DFC wrote—review & editing; AS, TI, and AC visualized the study; KU, DFC, and ME supervised the study; DFC involved in project administration; ME involved in funding acquisition.

### Peer review

The peer review history for this article is available at https://publons.com/publon/10.1002/1878‐0261.13135.

## Supporting information


**Fig. S1**. Region of interest selection and computer‐aided detection of Ki67 positive cells.
**Fig. S2**. Upregulation of COX2 protein levels in *PIK3CA*‐induced neoplastic lesions shows a vascular distribution.
**Fig. S3**. Morphological and histochemical evidence of increased lipids in *PIK3CA*‐induced neoplastic lesions.
**Fig. S4**. Stellate cells in *PIK3CA* mutant form injection‐induced lesions.
**Fig. S5**. Upregulation of *PIK3CA* canonical effectors in *PIK3CA* mutant form stably transfected HCC cell lines.
**Fig. S6**. Enhanced sensitivity of *PIK3CA* mutant form stably transfected HCC cell lines to Alpelisib.
**Fig. S7**. Combination indices of Alpelisib and OTX008 treatments in *PIK3CA* mutant form stably transfected HCC cell lines.
**Fig. S8**. PLC/PRF/5 cells treated with different PI3K inhibitors and OTX008 in mono‐ or combination therapy for 72 h.
**Fig. S9**. Drug screening of 315 approved anti‐cancer compounds with and without OTX008.
**Fig. S10**. Microarray analysis of *PIK3CA* E545K and H1047R effectors in stably transfected HCC cell lines SNU387 and SNU449.
**Fig. S11**. Differential effectors of *PIK3CA* E545K and H1047R in stably transfected HCC cell lines SNU387 and SNU449.
**Fig. S12**. Gene Set Enrichment Analysis of *PIK3CA* E545K and H1047R in stably transfected HCC cell lines SNU387 and SNU449.

**Table S1**. Overview of drug targets with ≥ 1 hit.

**Table S2**. Combinatorial drug screening results for PLC/PRF/5 cells.

## Data Availability

The gene expression microarray data that support the findings of this study are openly available in NCBI's Gene Expression Omnibus and are accessible through https://www.ncbi.nlm.nih.gov/geo/query/acc.cgi?acc=GSE173963, GEO Series accession number GSE173963 (mouse liver lysate comparisons) and https://www.ncbi.nlm.nih.gov/geo/query/acc.cgi?acc=GSE182915, GEO Series accession number GSE182915 (HCC cell lines comparisons).
