# Supplementary material for: RASSF1A independence and early galectin‐1 upregulation in PIK3CA‐induced hepatocarcinogenesis: new therapeutic venues
Source: Mol Oncol. 2021 Nov 20;16(5):1091–118. doi: 10.1002/1878-0261.13135 (PMC8895452; doi:10.1002/1878-0261.13135)
Supplement: Supplementary file 1 — Fig. S1. Region of interest selection and computer‐aided detection of Ki67 positive cells. Fig. S2. Upregulation of COX2 protein levels in PIK3CA‐induced neoplastic lesions shows a vascular distribution. Fig. S3. Morphological and histochemical evidence of increased lipids in PIK3CA‐induced neoplastic lesions. Fig. S4. Stellate cells in PIK3CA mutant form injection‐induced lesions. Fig. S5. Upregulation of PIK3CA canonical effectors in PIK3CA mutant form stably transfected HCC cell lines. Fig. S6. Enhanced sensitivity of PIK3CA mutant form stably transfected HCC cell lines to Alpelisib. Fig. S7. Combination indices of Alpelisib and OTX008 treatments in PIK3CA mutant form stably transfected HCC cell lines. Fig. S8. PLC/PRF/5 cells treated with different PI3K inhibitors and OTX008 in mono‐ or combination therapy for 72 h. Fig. S9. Drug screening of 315 approved anti‐cancer compounds with and without OTX008. Fig. S10. Microarray analysis of PIK3CA E545K and H1047R effectors in stably transfected HCC cell lines SNU387 and SNU449. Fig. S11. Differential effectors of PIK3CA E545K and H1047R in stably transfected HCC cell lines SNU387 and SNU449. Fig. S12. Gene Set Enrichment Analysis of PIK3CA E545K and H1047R in stably transfected HCC cell lines SNU387 and SNU449. [file MOL2-16-1091-s003.docx]

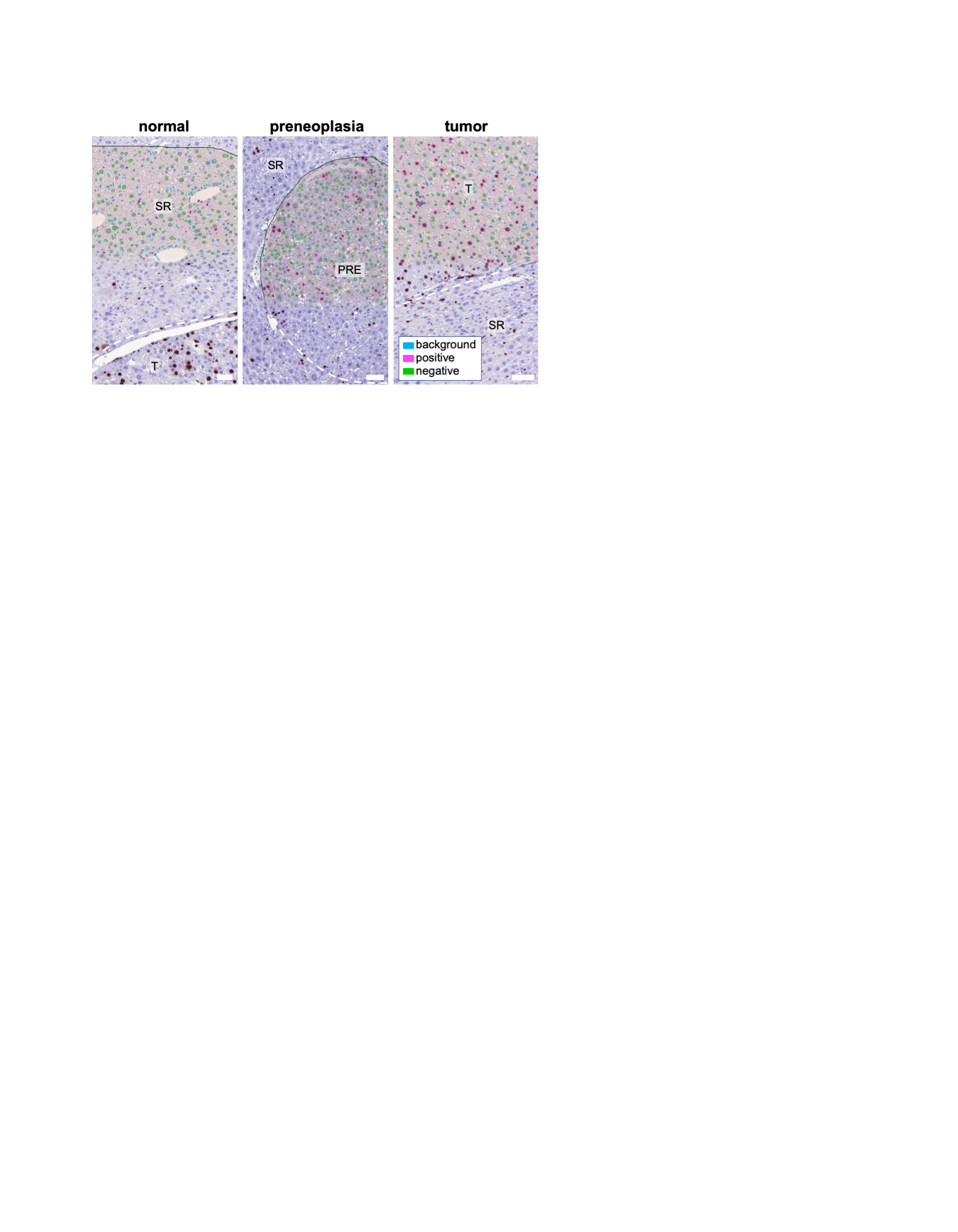


Figure S1. *Region of interest selection and computer-aided detection of Ki67 positive cells.* Screenshots obtained from DeePathology™STUDIO software exemplifying the region of interest selection (orange shading) and the detected nuclei with attribution of the properties background, positive or negative. Transparency gradient to original images without overlay from top to bottom. *Scale bars*: 50 µm. T, tumor; PRE, preneoplastic lesion; SR, surrounding tissue.


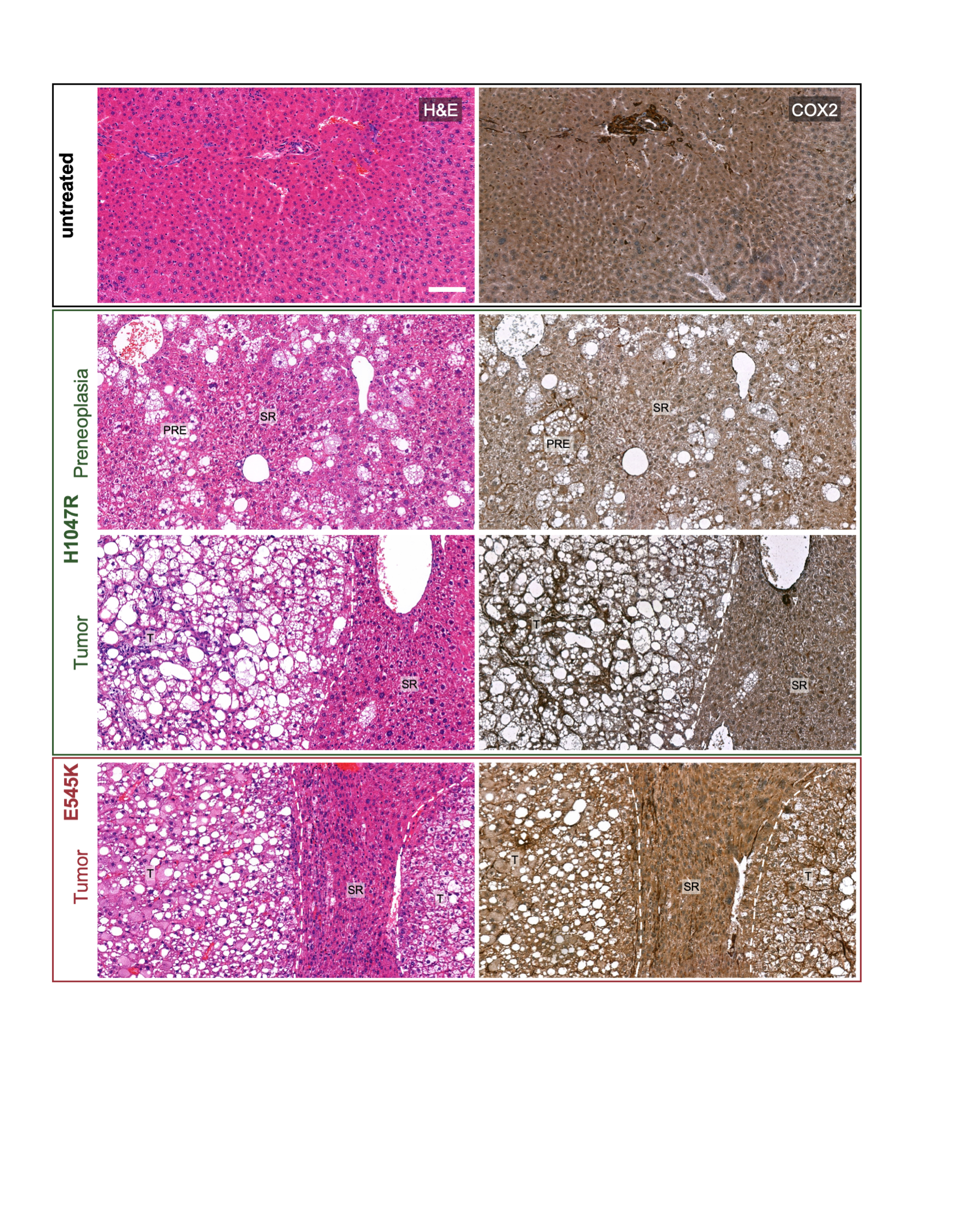


Figure S2. *Upregulation of COX2 protein levels in PIK3CA-induced neoplastic lesions shows a vascular distribution.* Representative immunohistochemical analyses of PIK3CA induced preneoplastic lesions and tumors (*right panels*) with corresponding H&E (*left panels*) highlight an increased intralesional vascular expression of COX2. *Scale bar*: 100 µm. COX2, Prostaglandin-endoperoxide synthase 2; T, tumor; SR, surrounding tissue.


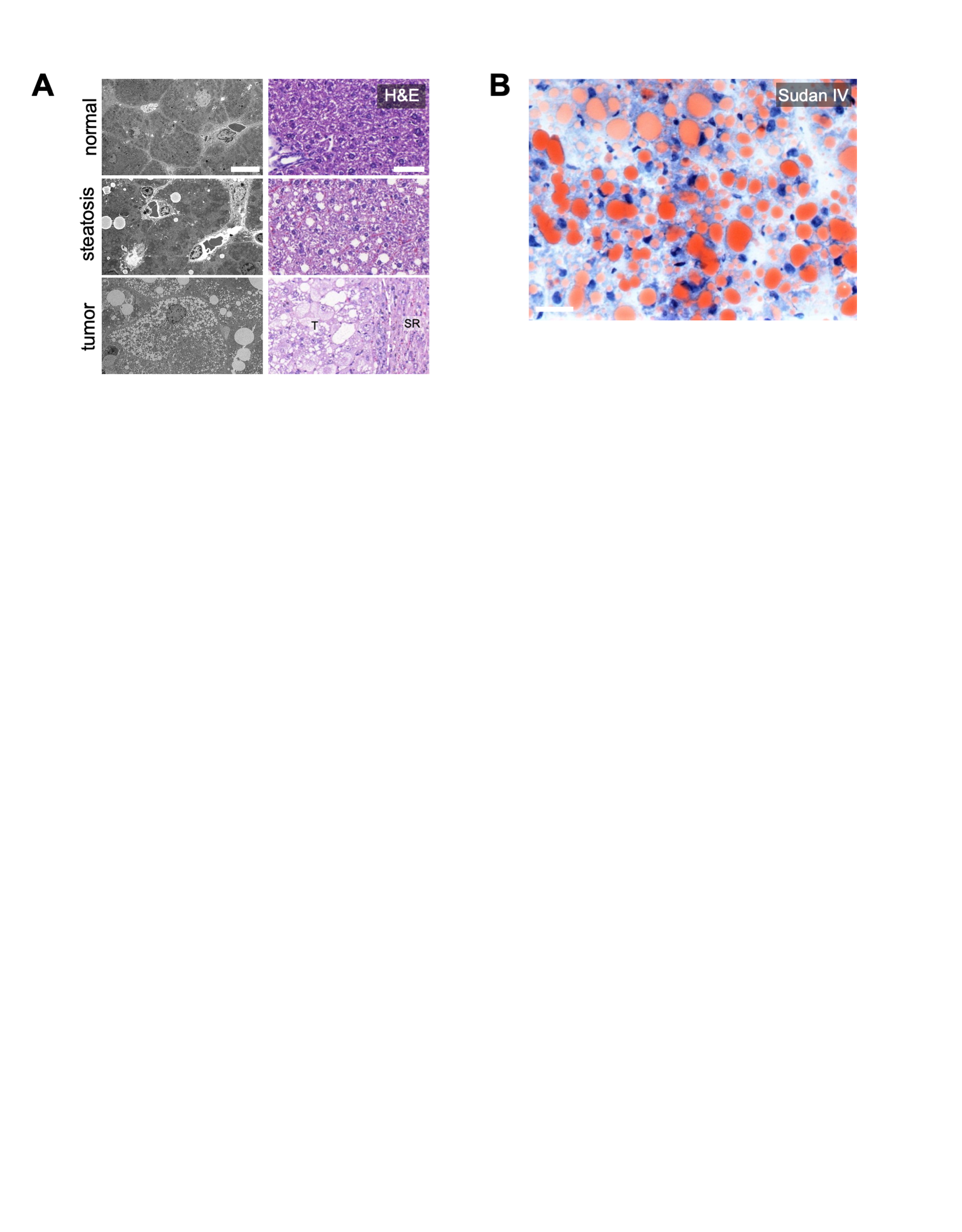


Figure S3. *Morphological and histochemical evidence of increased lipids in PIK3CA-induced neoplastic lesions. (A)* Transmission electron micrographs of normal liver tissue of an untreated mouse at the experimental timepoint of 1 week (*upper panel*), an untreated mouse at the 6 months time point with macrovesicular steatosis (*middle panel*), and a *PIK3CA* E545K induced tumor (*lower panel*) at a 9 months time point with diffuse microvesicular lipid inclusions. Adjacent matched H&E stained histological sections. *Scale bars*: 10 µm (electron micrographs), 50 µm (histological sections). (*B*) Histochemical Sudan IV staining of a *PIK3CA* E545K injected tumor highlighting cytoplasmic lipid droplets. *Scale bar*: 50 µm. T, tumor; SR, surrounding tissue.


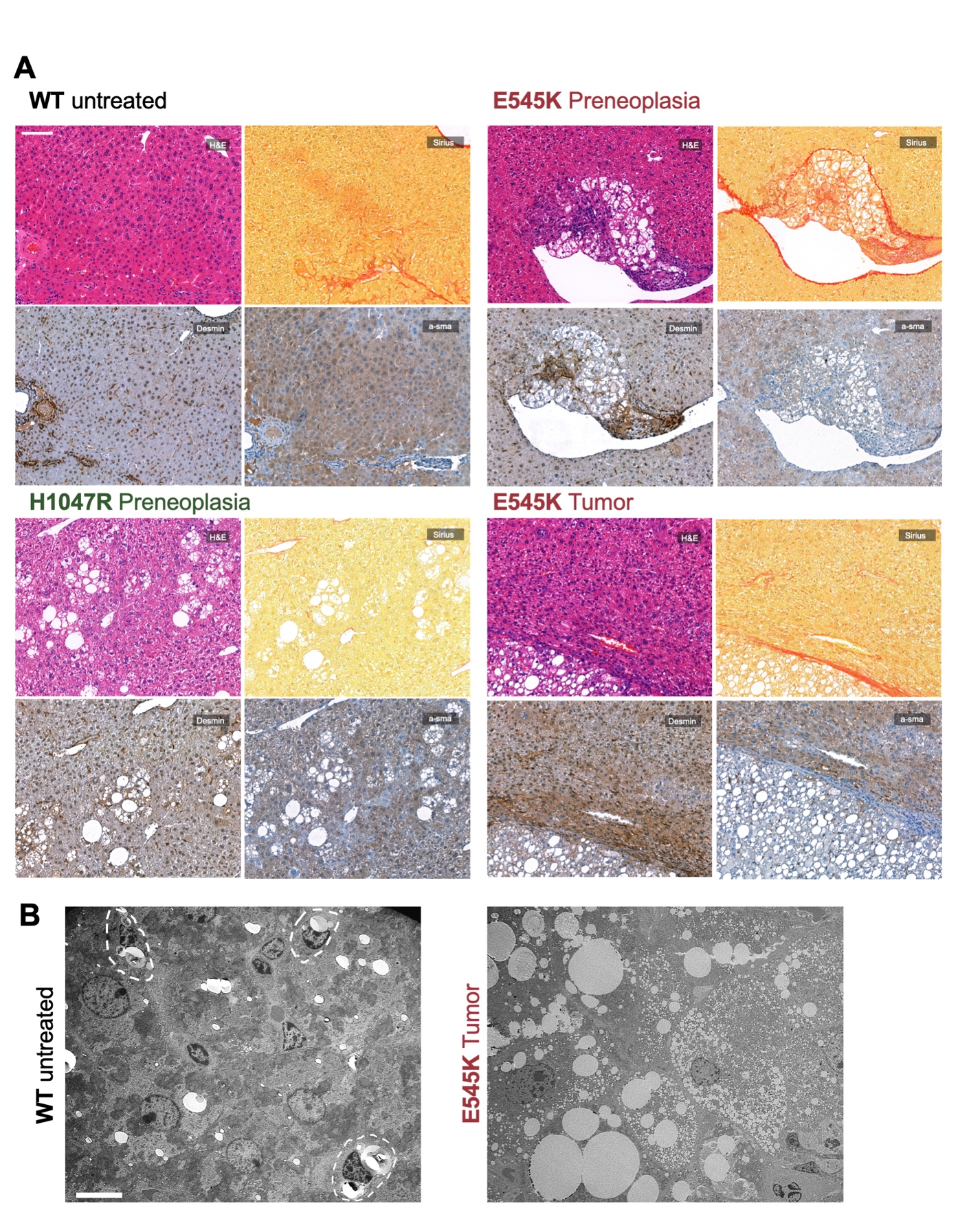


Figure S4. *Stellate cells in PIK3CA mutant injection induced lesions. (A)* Representative histological images of untreated liver tissue, a *PIK3CA* E545K induced preneoplastic lesion, *PIK3CA* H1047R induced preneoplastic lesions and a *PIK3CA* E545K induced tumor. Picrosirius red staining (Sirius) demonstrates the absence of increased fibrosis in preneoplastic and neoplastic lesions compared to wildtype untreated mice. Stellate cells neither increase in number (desmin-immunohistochemistry) nor do they display pronounced activation as observed by alpha-smooth muscle actin (a-sma) in preneoplastic and neoplastic lesions when compared to untreated wildtype mice. *Scale bar*: 100 µm. (B) Representative transmission electron micrographs of normal liver tissue of an untreated mouse (*left panel*) and a tumor in a *PIK3CA* E545K injected mouse (*right panel*) with stellate cells highlighted by a dashed line, demonstrating the scarcity/absence of stellate cells in the induced lesions. *Scale bar*: 10 µm.


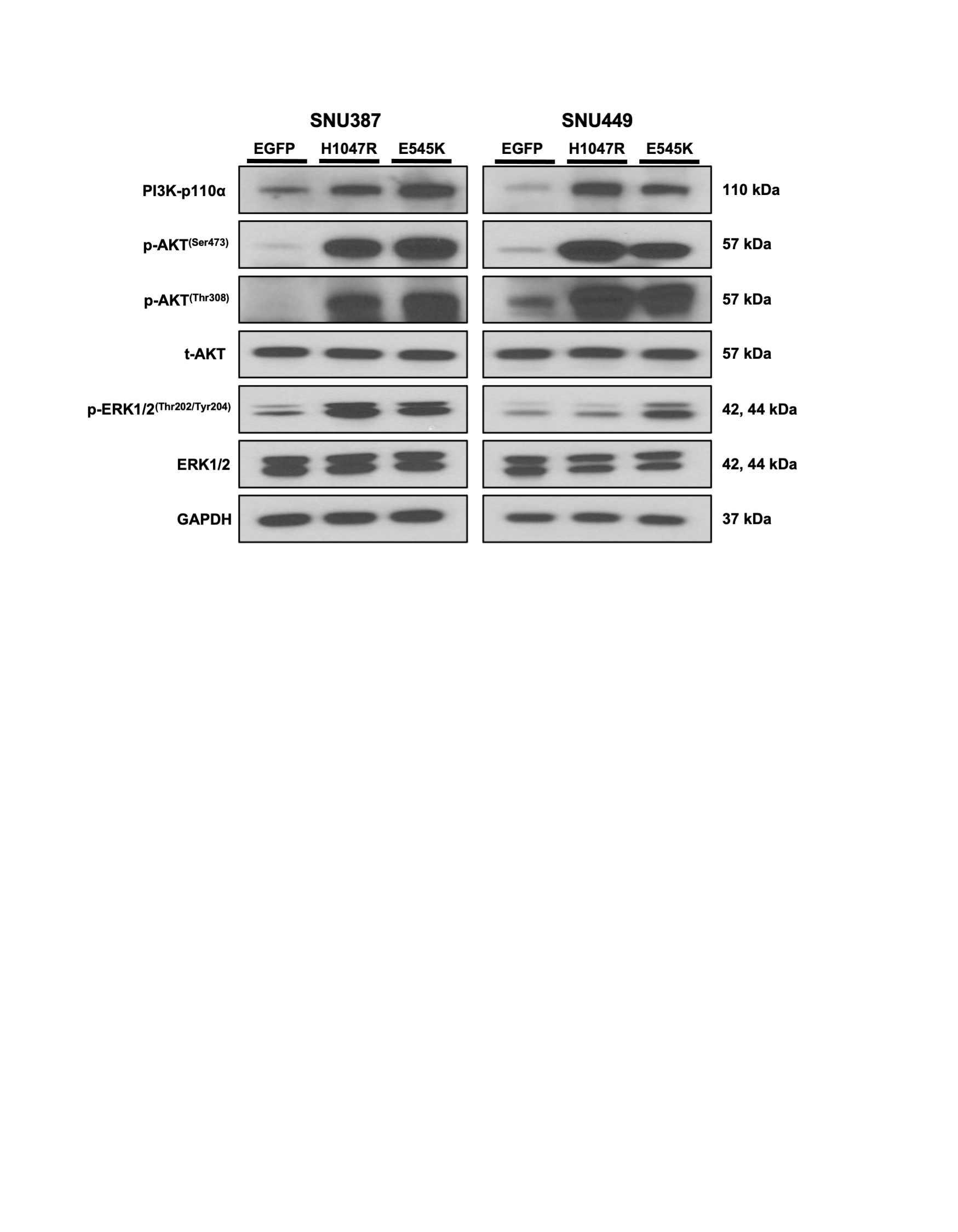


Figure S5. *Upregulation of PIK3CA canonical effectors in PIK3CA mutant form stably transfected HCC cell lines.* Western blot analysis of PI3K-p110α and its downstream effectors p-AKT (Ser473), p-AKT (Thr308), p-ERK1/2 (Thr202, Tyr204) in SNU387 and SNU449 HCC cell lines stably transfected with EGFP, PIK3CA H1047R, or PIK3CA E545K constructs with corresponding total protein levels of AKT and ERK1/2. Loading control: GAPDH. Molecular weights of observed bands are marked on the right. (p)ERK1/2, (phosphorylated) extracellular-signal-regulated kinases 1/2; (p/t)AKT, (phosphorylated/total) RAC-alpha serine/threonine-protein kinase; GAPDH, glyceraldehyde 3-phosphate dehydrogenase; PI3K-p110α, phosphatidylinositol 3-kinase p110α; EGFP, enhanced green fluorescent protein.


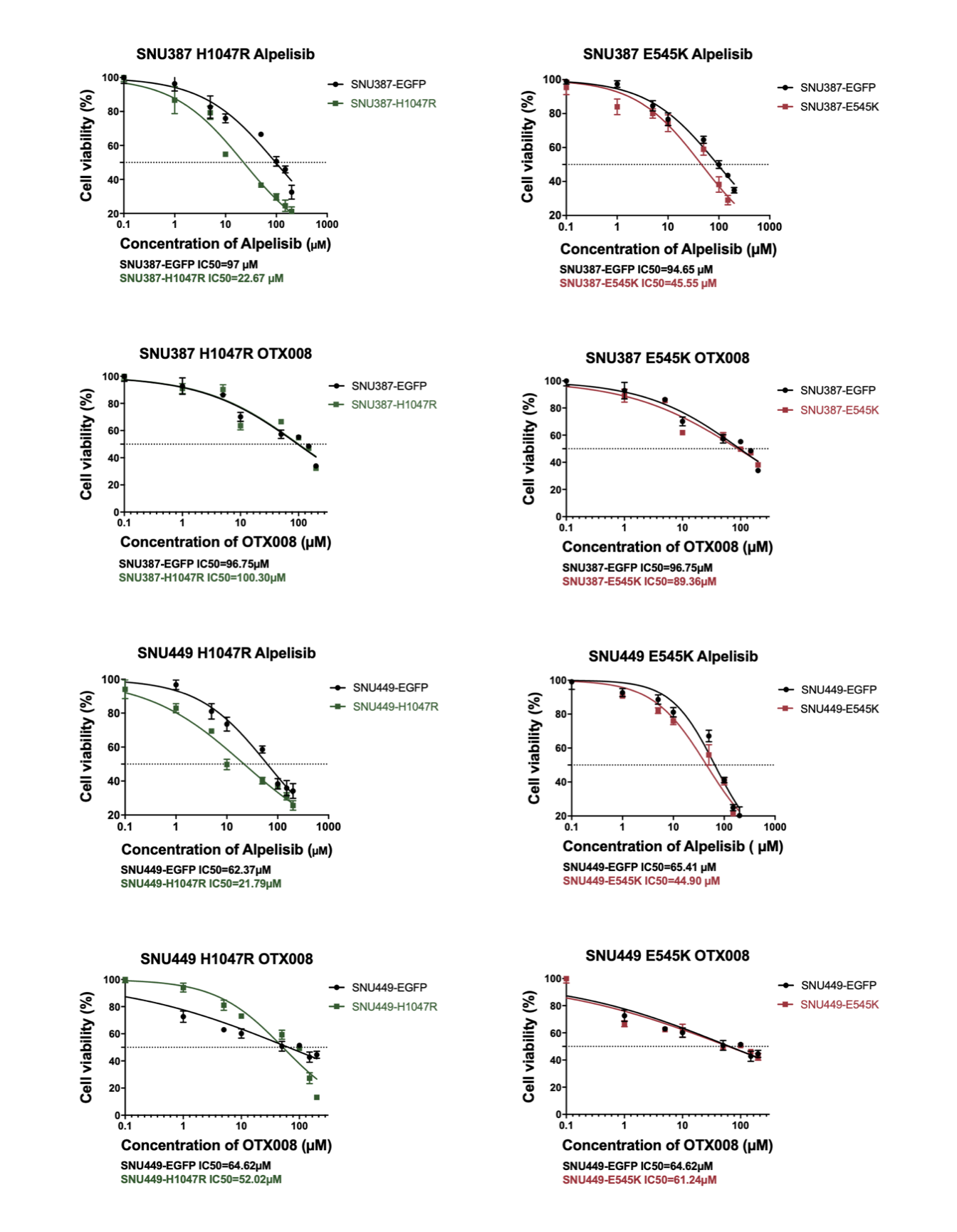


Figure S6. *Enhanced sensitivity of PIK3CA mutant form stably transfected HCC cell lines to Alpelisib.* Graphs depicting the cell viability as obtained from triplicate measurements in crystal violet assays of HCC cell lines SNU387 and SNU449 stably transfected with EGFP, *PIK3CA* H1047R, and *PIK3CA* E545K constructs after 48 hours of Alpelisib or OTX008 treatment. A decrease in IC50 for Alpelisib can be observed in all the *PIK3CA* mutant forms stably transfected cell lines, while the IC50 for OTX008 remains unaffected by transfection status. Data presented as mean with standard deviation. IC50, half-maximal inhibitory concentration.


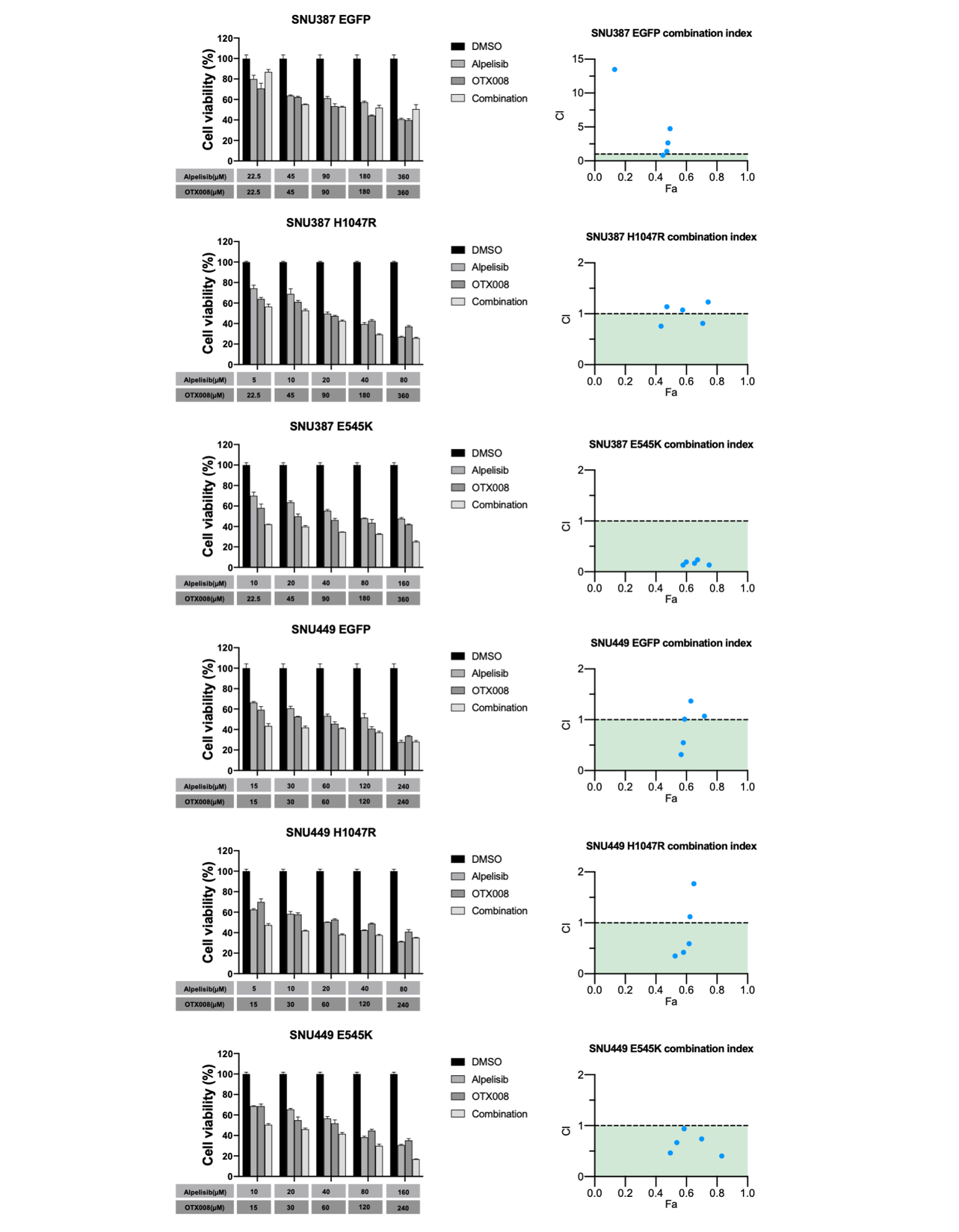


Figure S7. *Combination indices of Alpelisib and OTX008 treatments in HCC cell lines stably transfected with PIK3CA mutant forms.* Graphs depicting the cell viability as obtained from triplicate measurements in crystal violet assays of *EGFP*, *PIK3CA* H1047R, and *PIK3CA* E545K stably transfected HCC cell lines SNU387 and SNU449 after 48 hours of Alpelisib-, OTX008- or combined treatments. Data are presented as mean with standard deviation. Corresponding combination indices are shown on the right. A combination index (CI) < 1 is interpreted as synergy, while combination index values of < 1 (colored in green), 1, >1 can be interpreted as synergistic, additive, and antagonistic effects, respectively. While the effect of Alpelisib and OTX008 is even antagonistic in the SNU387 EGFP transfected cell line, PIK3CA mutant form stable transfection favors synergy. However, the effect is less apparent in SNU449 stably transfected cell lines.


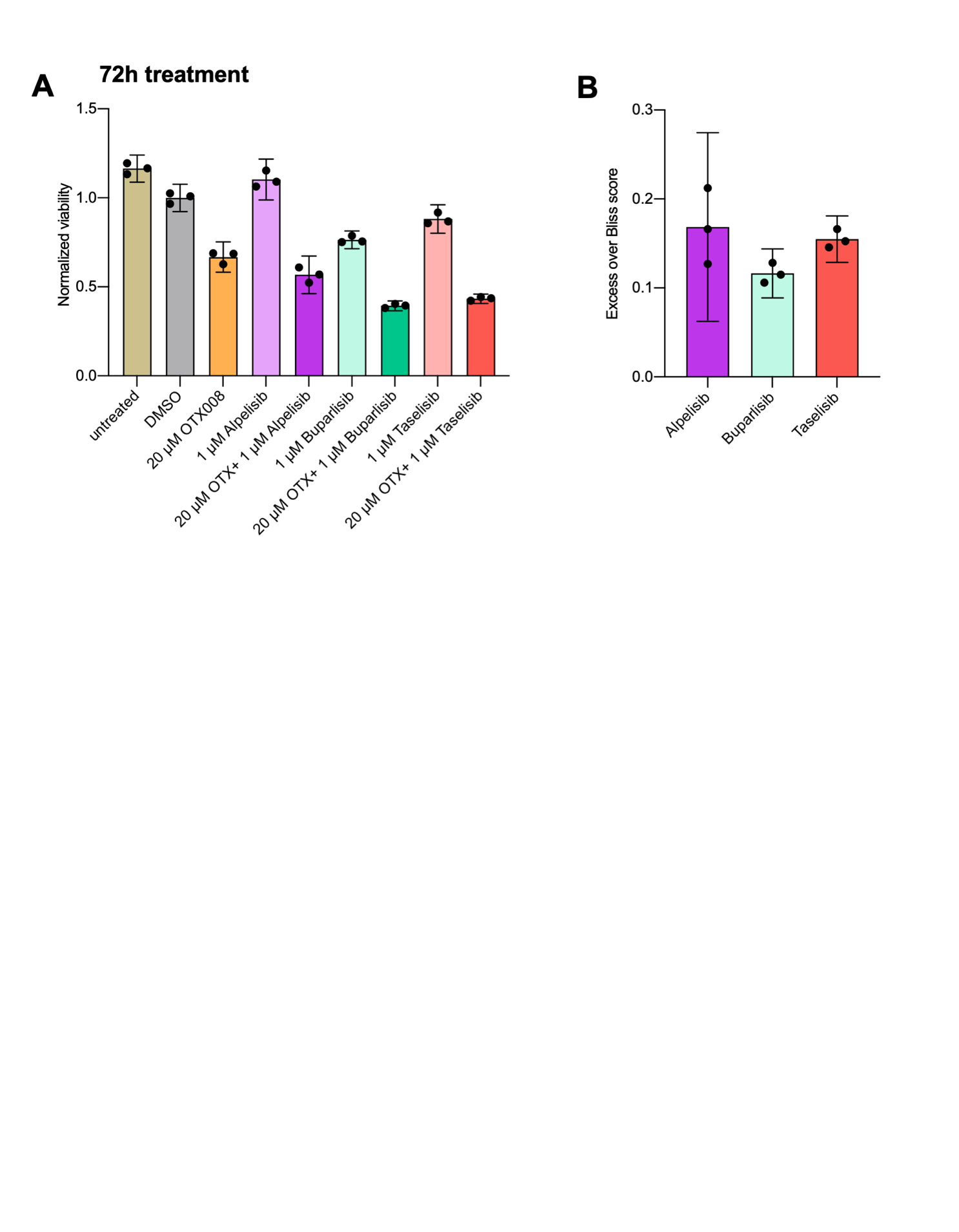


Figure S8. *PLC/PRF/5 cells treated with different PI3K inhibitors and OTX008 in mono- or combination therapy for 72 hours.* (*A*) Bar graph showing viability measured by ATP content normalized to mean of DMSO treated cells. (*B*) Bar graph showing the excess over the Bliss independence model for each of the PI3K inhibitors combined with OTX008. Data are shown as mean with 95% confidence interval.


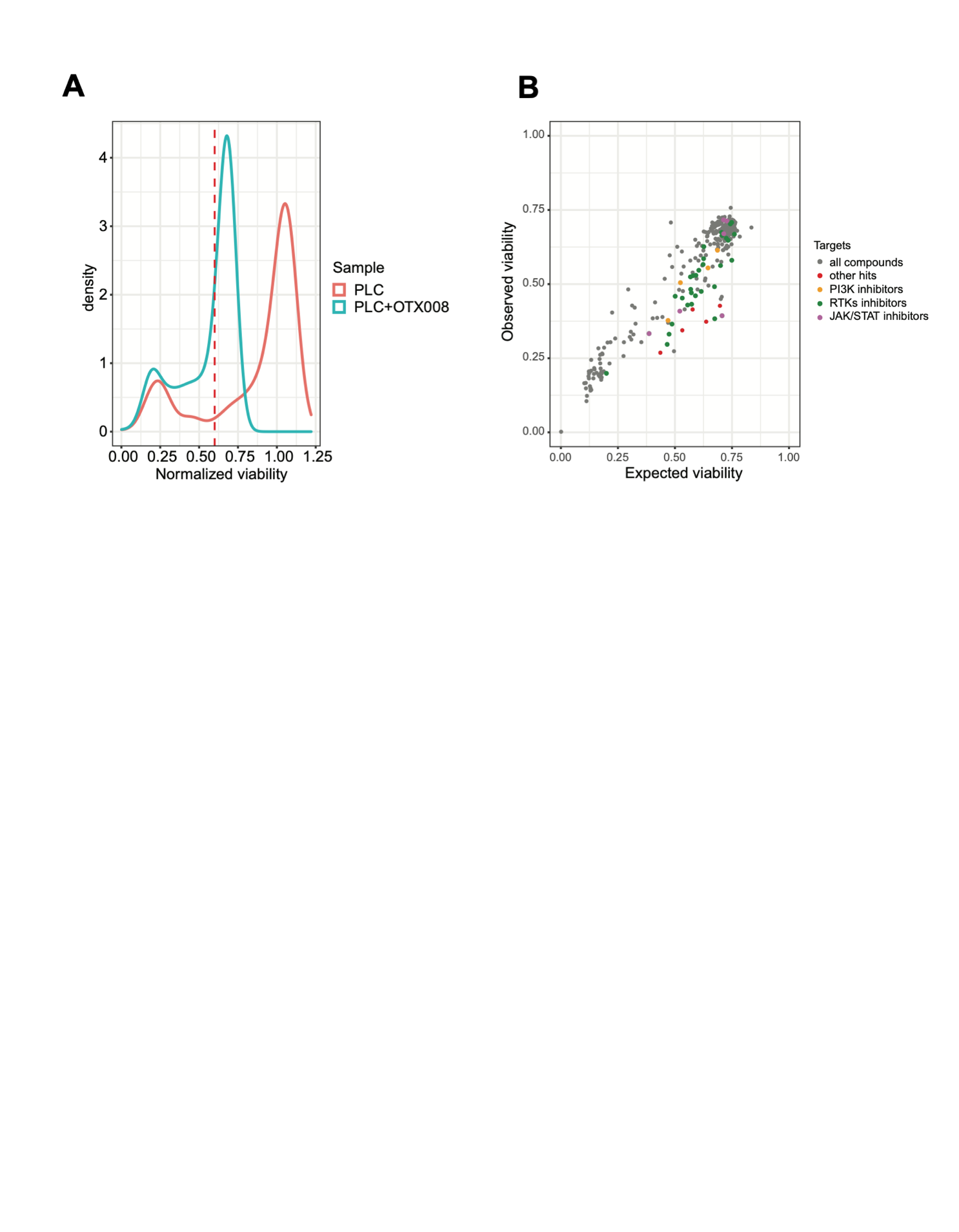


Figure S9. *Drug screening of 315 approved anti-cancer compounds with and without OTX008.* (*A*) Density plot of normalized viability data obtained from the screening of PLC/PRF/5 cells against a library of 315 approved anti-cancer compounds. Viability, measured as the number of Hoechst 33342 stained nuclei, is normalized to cells treated with DMSO only. The two conditions, with and without OTX008 1 µM, are shown separately. The threshold of normalized viability = 0.6 is shown as a dashed red line (one of the parameters for a compound to be considered as a hit). (*B*) Scatter plot of observed normalized viability from the combinatory screening against the expected viability calculated from the measured normalized viability of single treatment according to the Bliss independence model. Compounds showing normalized viability in the combination therapy < 0.6 and Z score of Excess over Bliss score of > 1.5 are defined as hits. Compounds targeting specific pathways are highlighted in respective colors: PI3K inhibitors in orange, tyrosine kinase inhibitors (RTKs inhibitors) in green, and JAK/STAT inhibitors in violet.


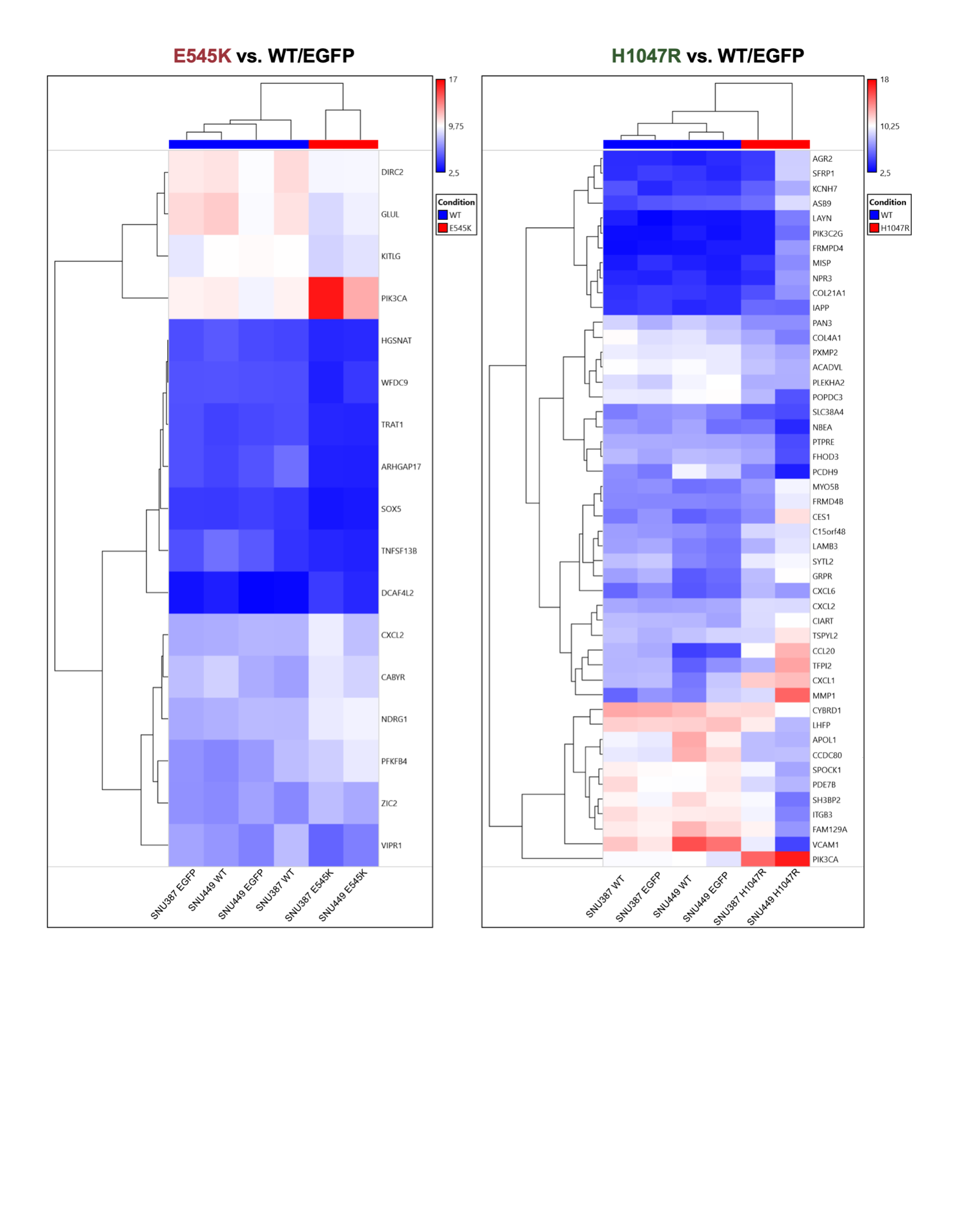


Figure S10. *Microarray analysis of PIK3CA E545K and H1047R effectors in stably transfected HCC cell lines SNU387 and SNU449.* (*A*) Heatmap of differentially expressed genes with selection criteria fold-change > 2, p < 0.05. For the analysis, EGFP transfected and wild type (WT) SNU387 and SNU449 were combined into one comparison group, and *PIK3CA* E545K transfected SNU387 and SNU449 samples were combined into another comparison group. (*B*) Heatmap of differentially expressed genes with selection criteria fold-change > 3, p < 0.05. For the analysis, EGFP transfected and wild type (WT) SNU387 and SNU449 were combined into one comparison group, and *PIK3CA* H1047R transfected SNU387 and SNU449 samples were combined into another comparison group. Gradient scale color codes for fold-changes. A cluster dendrogram is shown on the left and above.


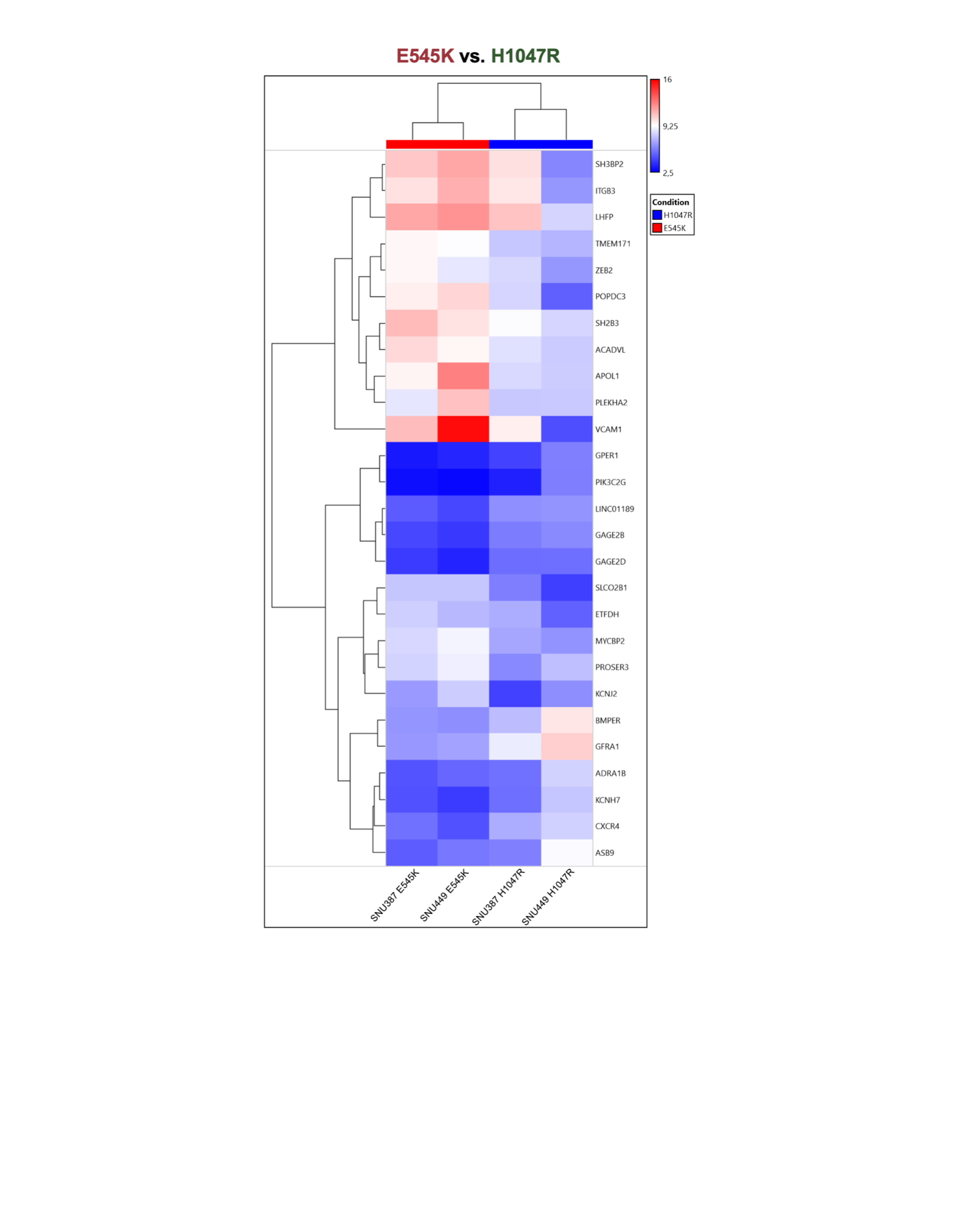


Figure S11. *Differential effectors of PIK3CA E545K and H1047R in stably transfected HCC cell lines SNU387 and SNU449.* Heatmap of differentially expressed genes with selection criteria fold-change > 3, *p* < 0.05. For the analysis, *PIK3CA* E545K transfected SNU387 and SNU449 cell lines were compared against *PIK3CA* H1047R transfected SNU387 and SNU449 cell lines. Gradient scale color codes for fold-changes. A cluster dendrogram is shown on the left and above.


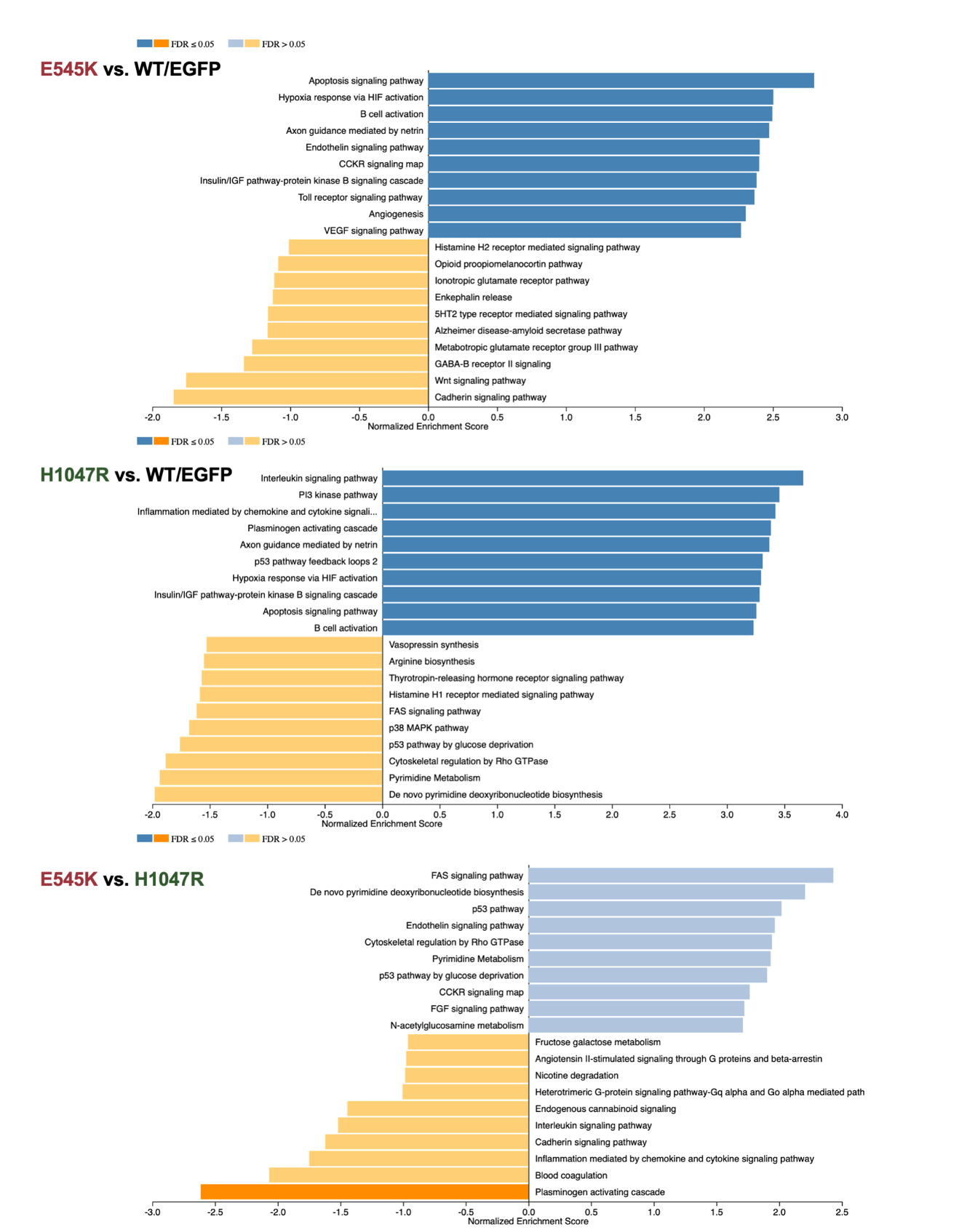


Figure S12. *Gene Set Enrichment Analysis of PIK3CA E545K and H1047R in stably transfected HCC cell lines SNU387 and SNU449.* GSEA was performed to identify significantly (i.e., false discovery rate (FDR) < 0.05) upregulated (dark blue) or downregulated (dark orange) pathways in *PIK3CA* E545K transfected HCC cell lines versus WT/ EGFP cell lines (upper graph), in *PIK3CA* H1047R transfected HCC cell lines versus WT/ EGFP cell lines (middle graph), and in *PIK3CA* H1047R versus *PIK3CA* E545K transfected SNU387 and SNU449 cell lines (lower graph). The Panther pathway gene set was employed.
