# Supplementary material for: RASSF1A independence and early galectin‐1 upregulation in PIK3CA‐induced hepatocarcinogenesis: new therapeutic venues
Source: Mol Oncol. 2021 Nov 20;16(5):1091–118. doi: 10.1002/1878-0261.13135 (PMC8895452; doi:10.1002/1878-0261.13135)
Supplement: Supplementary file 2 — Table S1. Overview of drug targets with ≥ 1 hit. [file MOL2-16-1091-s001.docx]

**Table S1:** Overview of drug targets with ≥ 1 hit

| Target | Hits | Non hits | All | Frequency |
| --- | --- | --- | --- | --- |
| COMT | 1.00 | 0.00 | 1.00 | 100.00 |
| CSF1 | 1.00 | 0.00 | 1.00 | 100.00 |
| CSK | 1.00 | 0.00 | 1.00 | 100.00 |
| FGFR4 | 1.00 | 0.00 | 1.00 | 100.00 |
| HIF1A | 1.00 | 0.00 | 1.00 | 100.00 |
| PLK4 | 1.00 | 0.00 | 1.00 | 100.00 |
| FGFR2 | 2.00 | 1.00 | 3.00 | 66.67 |
| CYP1A1 | 1.00 | 1.00 | 2.00 | 50.00 |
| CYP1B1 | 1.00 | 1.00 | 2.00 | 50.00 |
| FGFR3 | 2.00 | 2.00 | 4.00 | 50.00 |
| INSR | 1.00 | 1.00 | 2.00 | 50.00 |
| JAK3 | 1.00 | 1.00 | 2.00 | 50.00 |
| FLT3 | 4.00 | 6.00 | 10.00 | 40.00 |
| FGFR1 | 3.00 | 5.00 | 8.00 | 37.50 |
| KDR | 6.00 | 11.00 | 17.00 | 35.29 |
| DDR2 | 1.00 | 2.00 | 3.00 | 33.33 |
| ERBB2 | 2.00 | 4.00 | 6.00 | 33.33 |
| ERBB4 | 1.00 | 2.00 | 3.00 | 33.33 |
| FLT1 | 4.00 | 8.00 | 12.00 | 33.33 |
| MET | 1.00 | 2.00 | 3.00 | 33.33 |
| POLD1 | 1.00 | 2.00 | 3.00 | 33.33 |
| FLT4 | 4.00 | 9.00 | 13.00 | 30.77 |
| ALK | 1.00 | 3.00 | 4.00 | 25.00 |
| JAK1 | 1.00 | 3.00 | 4.00 | 25.00 |
| JAK2 | 1.00 | 3.00 | 4.00 | 25.00 |
| PDGFRA | 3.00 | 9.00 | 12.00 | 25.00 |
| PDGFRB | 4.00 | 12.00 | 16.00 | 25.00 |
| POLE | 1.00 | 3.00 | 4.00 | 25.00 |
| RRM2B | 1.00 | 3.00 | 4.00 | 25.00 |
| KIT | 4.00 | 13.00 | 17.00 | 23.53 |
| EGFR | 3.00 | 10.00 | 13.00 | 23.08 |
| CSF1R | 2.00 | 7.00 | 9.00 | 22.22 |
| MAP2K1 | 1.00 | 4.00 | 5.00 | 20.00 |
| POLA1 | 1.00 | 4.00 | 5.00 | 20.00 |
| RAF1 | 1.00 | 4.00 | 5.00 | 20.00 |
| RET | 2.00 | 8.00 | 10.00 | 20.00 |
| BRAF | 1.00 | 5.00 | 6.00 | 16.67 |
| RRM1 | 1.00 | 5.00 | 6.00 | 16.67 |
| RRM2 | 1.00 | 5.00 | 6.00 | 16.67 |
| CYP19A1 | 1.00 | 6.00 | 7.00 | 14.29 |
| TUBB | 1.00 | 9.00 | 10.00 | 10.00 |
